# An integrated framework for discovery and genotyping of genomic variants from high-throughput sequencing experiments

**DOI:** 10.1093/nar/gkt1381

**Published:** 2014-01-11

**Authors:** Jorge Duitama, Juan Camilo Quintero, Daniel Felipe Cruz, Constanza Quintero, Georg Hubmann, Maria R. Foulquié-Moreno, Kevin J. Verstrepen, Johan M. Thevelein, Joe Tohme

**Affiliations:** ^1^Agrobiodiversity research area, International Center for Tropical Agriculture (CIAT), Km 17 Recta Cali- Palmira, A.A. 6713 Cali, Colombia, ^2^Laboratory of Molecular Cell Biology, Department of Biology, Institute of Botany and Microbiology, KU Leuven, Kasteelpark Arenberg 31, B-3001 Leuven-Heverlee, Flanders, Belgium, ^3^Department of Molecular Microbiology, VIB, Kasteelpark Arenberg 31, B-3001 Leuven-Heverlee, Flanders, Belgium, ^4^VIB Laboratory of Systems Biology, KU Leuven, Gaston Geenslaan 1, B-3001 Leuven-Heverlee, Flanders, Belgium and ^5^Laboratory for Genetics and Genomics, Centre of Microbial and Plant Genetics, KU Leuven, Gaston Geenslaan 1, B-3001 Leuven-Heverlee, Flanders, Belgium

## Abstract

Recent advances in high-throughput sequencing (HTS) technologies and computing capacity have produced unprecedented amounts of genomic data that have unraveled the genetics of phenotypic variability in several species. However, operating and integrating current software tools for data analysis still require important investments in highly skilled personnel. Developing accurate, efficient and user-friendly software packages for HTS data analysis will lead to a more rapid discovery of genomic elements relevant to medical, agricultural and industrial applications. We therefore developed Next-Generation Sequencing Eclipse Plug-in (NGSEP), a new software tool for integrated, efficient and user-friendly detection of single nucleotide variants (SNVs), indels and copy number variants (CNVs). NGSEP includes modules for read alignment, sorting, merging, functional annotation of variants, filtering and quality statistics. Analysis of sequencing experiments in yeast, rice and human samples shows that NGSEP has superior accuracy and efficiency, compared with currently available packages for variants detection. We also show that only a comprehensive and accurate identification of repeat regions and CNVs allows researchers to properly separate SNVs from differences between copies of repeat elements. We expect that NGSEP will become a strong support tool to empower the analysis of sequencing data in a wide range of research projects on different species.

## INTRODUCTION

Recent advances in high-throughput sequencing (HTS) technologies have allowed research groups to produce unprecedented amounts of genomics data that have been of great use in exploring the genetic variability among and within any kind of species and in determining the genetic causes of phenotypic variation. These technologies have been successfully applied to make significant discoveries in highly dissimilar research fields such as human genetics (1), cancer research (2), crop breeding (3) and even the industrial production of biofuels (4). One of the major bottlenecks in projects involving HTS is the bioinformatics capacity (in hardware, software and personnel) needed to analyze the large amounts of data produced by the technology and to deliver valuable information such as genes related to traits or diseases or markers for genomic selection. Because significant advances have been made in increasing computing capacity, the main reason for this bottleneck is that software packages for analysis of HTS data are still under development and any project involving HTS data requires close collaboration with trained bioinformaticians. The development of fast, accurate and easy-to-use software packages and analysis pipelines will empower scientists to perform by themselves the data analysis required to discover the genes, DNA elements or genomic variants related to their particular research interests.

In this work, we focus on the analysis pipeline required to discover genomic differences between a sequenced sample and a reference genome that is a representative DNA sequence assumed to be genetically close to the sample. In this case, samples are sequenced at moderate coverage (10× to 40× depending on genome length and heterozygosity) and then a generic bioinformatics pipeline aligns the reads to the reference sequence to find the most likely origin of each read in the genome. These alignments are then used to produce a catalog of genomic differences between the sample and the reference sequence (see an example schematic in Supplementary Figure S1). Several algorithms and software tools have been recently developed to resolve the different steps of this pipeline [see (5) and (6) for recent reviews]. Unfortunately, most of these tools require some sort of bioinformatics support to be operated and integrated, which is further complicated by the complexity of dealing with differences in programming languages, maintenance, efficiency, formats for data exchange, usability and even code quality. Commercial packages such as CLC Bioinformatics or Lasergene provide an alternative for solving this problem but at the expense of costly software licensing and limited capacity to perform nonstandard analysis.

Here, we describe Next-Generation Sequencing Eclipse Plug-in (NGSEP), a new integrated user-friendly framework for standard analysis of HTS reads. The main functionality of NGSEP is the variants detector, which allows researchers to make integrated discovery of single nucleotide variants (SNVs), small and large indels and regions with copy number variation (CNVs). NGSEP also provides a user interface for Bowtie 2 (7) to perform mapping to the reference genome and other utilities such as alignments sorting, merging of variants from different samples and functional annotation of variants. Using real sequencing data from yeast, rice and human samples we show that the algorithms implemented in NGSEP provide the same or better accuracy and efficiency than the recently published algorithms GATK (8,9), SAMtools (10), SNVer (11), VarScan 2 (12,13), CNVnator (14) and BreakDancer (15). We also compared the results of SNV and CNV detection for different read alignment strategies implemented in the packages BWA (16) and Bowtie 2 (7). NGSEP is distributed as an open-source java package available at https://sourceforge.net/projects/ngsep/.

## MATERIALS AND METHODS

### Data sets

We downloaded high-coverage sequencing reads for the CEU individual NA12878 from the pilot project of the 1000 Genomes Consortium currently available at ftp://ftp.1000genomes.ebi.ac.uk/vol1/ftp/pilot_data/data/. Low-coverage data were also downloaded from the first release of the 1000 genomes project (ftp://ftp.1000genomes.ebi.ac.uk/vol1/ftp/data/). Yeast samples were sequenced by the group of Johan Thevelein as part of an effort to find genes sustaining low glycerol production [see (4) for details].

Rice seeds of IR8 (accession BCF 941) were planted in the greenhouse facility at CIAT (International Center for Tropical Agriculture). Genomic DNA was prepared from a single plant as follows: 1 g of leaf tissue of a 45-DAP seedling was collected and ground with liquid nitrogen. DNA was isolated according to the urea-phenol extraction protocol modified from (17). DNA quality was tested before whole-genome sequencing so that the concentration exceeded 500 ng/μL and the A260/280 ratio was 1.8. DNA was sequenced on the Illumina HiSeq 2000 by the Yale Center for Genome Analysis (http://medicine.yale.edu/keck/ycga/index.aspx).

### Description of algorithms implemented in NGSEP

We built NGSEP based on the previously published software NGSTools (18). We redesigned parts of the initial package to improve its performance and we also fixed a few errors found by original users of NGSTools. As discussed in the ‘Results’ section, we included one parameter to control the maximum number of reads starting at each reference site and another parameter to control the maximum value of a base quality score. To perform realignment around indels, each time NGSEP detects a site with the start of an indel call it screens a few base pairs ahead (five for insertions, the length of the deletion plus two for deletions) to check whether the same indel is present in other reads at a different start site. If that is the case, the start site with the highest read support is chosen as the most likely start site and NGSEP changes the CIGAR field of alignments with indels starting at a site different from the chosen start.

We implemented the CNVnator algorithm as described in (14). This algorithm performs a statistical segmentation of the read depth (RD) pattern to identify regions with significant deviation from the average RD in single-copy regions. In the general literature, these types of algorithms are called read depth (RD) algorithms to contrast them with algorithms based on read pair (RP) data (19). Our implementation of CNVnator has four main differences compared with the algorithm as described in the article:

1. We created a parameter for the genome size.
2. We took only bins with unique alignments for the calculation of mean and variance of RD.
3. While merging small neutral regions between CNVs we check that the final region has a *P*-value below the minimum threshold.
4. Additional deletions calculated with the Gaussian method cannot override deletions called by the standard method.

RD approaches for CNV discovery rely on the assumption that the coverage is evenly distributed across the DNA present in the sample and hence regions with significant changes in the average RD become candidates for dosage altering variation. It has been observed that this assumption can be violated in at least two different ways (20). First, regions with extreme values of GC-content tend to be sequenced at lower coverages, which creates a need for an initial step of GC-correction of the read intensities. For NGSEP, we implemented the same strategy for GC-content correction implemented in CNVnator. Second, different treatments of reads with multiple alignments in the reference genome produce different biases in the estimation of the number of copies for each region (21). In most of the previous studies the initial data set contains only the best alignment for each read. For reads with two or more equally good alignments, one alignment is chosen at random (14). To account for RD biases, a ‘mappability score’ can be defined for each site of the genome taking for example the average mapping quality of the alignments spanning such site. Then, normalization can be performed by clustering sites with similar score and correcting the read intensities by applying the same procedure used to correct for GC-content biases (20).

Normalizing RD by mappability seeks to equalize the RD distribution on repetitive and unique regions. However, a repetitive region can be thought to be a region with a copy number different from the one of a unique region in the genome. Instead of trying to normalize the RD for such regions, we tried to use the RD to identify them. We believe that this could be particularly useful for draft genomes in which annotation of repetitive elements is still in progress. To accomplish this, we calculated the distribution of RD taking only the sites of the genome without ambiguously aligned reads (e.g. sites with good mappability scores). This allowed us to use Bowtie 2 to build different read alignment data sets for the yeast samples and then test the following strategies for the management of reads with multiple alignments: (i) pick the best alignment for each read (default and similar to BWA), (ii) keep up to three alignments per read and (iii) output all alignments found for each read. In the third approach, all regions with variable copy number, including known repeat elements are supposed to stand out from neutral regions. We tried the second option as an intermediate stage suitable for the analysis of samples with large genomes.

Because it has been shown that RD and RP approaches are complementary for detecting large deletions (22), we also implemented an RP algorithm for detecting large indels. As described in previous works (15,23,24), NGSEP walks over the reads sorted by genomic location and clusters unique overlapping alignments of RPs for which the predicted insert length is similar among them and deviates significantly from the average. While clusters with insert length larger than the average are used as evidence for detecting large deletions, clusters with insert length smaller than the average become evidence for large insertions. The length of each indel event is estimated as the average insert length of the alignments in the cluster supporting the event minus the average length of the whole data set. To calculate the significance of a cluster as a predictor of a large indel, we use the Poisson model described in the algorithm BreakDancerMax (15). This approach can also be used to detect other types of variation such as inversions, translocations and tandem duplications (25,26).

The source code of NGSEP implementing the algorithms described above is available as an open-source package under a GPL license (at https://sourceforge.net/projects/ngsep/).

### Construction of gold-standard genotypes for a yeast pool of segregants

Deep sequencing of two haploid yeast parents at an average coverage close to 80× allowed us to build a gold-standard data set of expected genotypes for a pool of 20 *F*_1_ haploid segregants randomly selected from an initial pool of 257 segregants produced by independent crosses of the two parents (4) (Supplementary Figure S2A). We extracted from each haploid parent the sites of the genome in which only one allele is observed using both BWA and Bowtie 2 alignments and in which this allele is called by NGSEP, GATK, SAMtools and VarScan with quality (GQ field or QUAL column if GQ is not present) >90. For the haploid parent CBS6412, this procedure yielded 9 321 880 high-quality genotype calls to the reference allele and 53 455 high-quality genotype calls to the alternative allele. For the haploid parent ER7A, the numbers of high-quality genotype calls were 9 894 901 to the reference allele and 53 050 to the alternative allele. Then, we assumed that the 8 301 038 sites with the reference allele in both parents and the 38 279 sites with the alternative allele in both parents will look like homozygous sites in the pool. Conversely, we assumed that the 16 904 sites in which the high-quality genotypes of the two haploid parents differ become heterozygous sites in the pool in which the two alleles should appear in nearly equal proportions.

To test the degree to which genetic drift could invalidate these assumptions, we performed 100 000 simulation experiments as follows: given a heterozygous site, we pick the number of segregants with the reference allele *n_i_* from a binomial distribution with parameters *n* = 20 and *p* = 0.5. Then, we pick the total RD *d_i_* from a Poisson distribution centered in the median coverage. Finally, we pick the number of times the reference allele is observed from a binomial distribution with parameters *n* = *n_i_* and *p* = *n_i_*/20. The distribution of the reference allele frequency obtained in this simulation is consistent with the observed distribution based on allele counts at the sites predicted to be heterozygous (Supplementary Figure S2B). We also performed two additional simulations, one increasing the number of segregants in the pool to 200, and a second one assuming a reference allele frequency of 0.5 (e.g. removing the effect of genetic drift). Although we could observe that genetic drift effectively increases the variance in the proportion of the reference allele, the observed distribution of allele frequencies based on read counts and the fact that we did not observe fixation of one allele at any of the sites predicted as being heterozygous suggests that an accurate method to perform standard genotyping of a diploid individual should also be accurate for identifying homozygous and heterozygous sites in the pool. VCF files with the gold-standard genotypes for the yeast unselected pool are available at https://sourceforge.net/projects/ngsep/files/benchmark/.

### Measures for quality assessment of genotype calls

Given a set of genotype calls and gold-standard genotypes for one sample we counted the number of genotypes that were consistent and the number that were inconsistent between the genotype calls and the gold standard (sites not included in the gold-standard are not used in any of the following calculations). Genotyping errors can be classified into the following types: (i) homozygous sites called heterozygous, (ii) heterozygous sites called homozygous, (iii) homozygous reference sites called homozygous variant and (iv) homozygous variant sites called homozygous reference. Because we observed in the validation with yeast samples that errors of type 3 and type 4 were almost negligible, and for all methods they disappear at a minimum quality score of 20, we focused the validation on errors of type 1 and 2. Let *G_o_*,*G_e_* be the sets of homozygous and heterozygous sites in the gold standard and *C_o_*,*C_e_* be the sets of sites with homozygous and heterozygous calls in the test data set. In this notation, the number of type 1 errors is 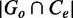 and the number of type 2 errors is 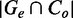. The following formulas summarize the calculations of sensitivity (*S*), false discovery rate (*FDR*) and false positive rate that we used to compare methods at different minimum quality thresholds and to build standard receiver operating characteristic (ROC) curves (27) comparing sensitivity and specificity:

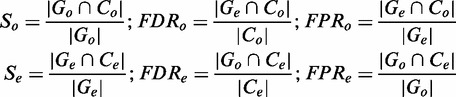



### Comparison with other tools

We assembled the GATK pipeline (version 2.7.2) as described on their best practices web page (http://www.broadinstitute.org/gatk/guide/best-practices), which includes the MarkDuplicates tool of Picard, indel realignment and quality score recalibration. Each command was executed with default parameters, except for the UnifiedGenotyper, for which we set the option stand_emit_conf to zero to maximize the calls produced by GATK and to be able to compare GATK genotypes with genotypes from other tools at different quality scores. For the yeast samples, we used the out mode EMIT_ALL_SITES to genotype every site of the genome. For the rice sample, we also set the prior heterozygosity rate to 0.0001. We called SNVs and small indels separately using the appropriate genotype likelihood model for each case. Because BAM files obtained from the 1000 Genomes Project were the result of the preprocessing pipeline of GATK (ftp://ftp.1000genomes.ebi.ac.uk/vol1/ftp/), we only ran the UnifiedGenotyper on those samples.

We ran SAMtools version 0.1.19 as suggested in the documentation for the mpileup command (http://samtools.sourceforge.net/mpileup.shtml). For the yeast samples we ran bcftools with the option -cg to retain every site covered in the genome. For VarScan (version 2.3.6) we ran the mpileup2cns tool as suggested in their documentation for germ line variants (http://varscan.sourceforge.net/germline-calling.html) to obtain genome-wide genotyping for the yeast samples. We compared the variants before and after applying the perl script for filtering described in the VarScan 2 paper (13). To facilitate independent replication of the results shown in this manuscript, we provide template scripts to run the different components of NGSEP, GATK, SAMtools and VarScan in a command line environment as supplementary material (Supplementary Scripts S1–S10).

We ran SNVer from its graphical interface using default parameters. Because SNVer did not provide a GQ field reporting the quality of genotype calls, we calculated the negative of the 10-based logarithm of the *P*-value as the quality score of their genotype calls. We ran CNVnator as suggested in the README included in the publicly available distribution (http://sv.gersteinlab.org/cnvnator/) using a window size of 100 bp. Finally, for BreakDancer we set the minimum mapping quality (q option) to 10. We performed all these experiments on a Dell computer with a quadruple Intel Xeon CPU at 2.93 GHz, 26.5 GB of available memory and 598.9 GB of total disk space.

## RESULTS

### Integrated variants discovery with NGSEP

**Figure 1.**
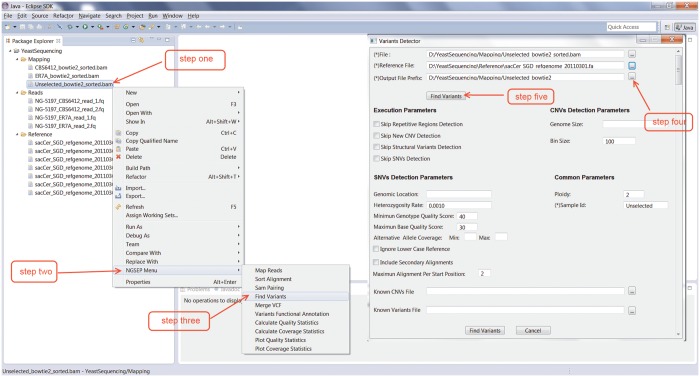
Common interaction with NGSEP to call variants from aligned reads. (i) Right-click on a sorted SAM or BAM file, (ii) select the menu for NGSEP, (iii) select the option to call variants, (iv) select the reference genome (only the first time) and a prefix for the output files and (v) click on the find variants button.

We developed the software package called NGSEP that integrates algorithms for discovery of SNVs, small and large indels and genomic regions with copy number variation (CNVs). We improved our recently published algorithm SNVQ for SNV detection (18), and we also expanded the model to allow discovery of small indels. For CNV discovery we built a new implementation of the algorithm CNVnator (14), one of the most accurate algorithms for detecting CNVs (6). Finally, we integrated a RP algorithm to detect large deletions and mid-size insertions as described in (15). To facilitate user interaction, we built a graphical interface based on the Eclipse infrastructure. Figure 1 shows a common user interaction to call variants with NGSEP. We implemented several features to enable users to run complete analysis of their samples and obtain VCF files with genotype calls starting from raw reads (Supplementary Table S1). VCF files produced by NGSEP can be exported into the input formats required by commonly used bioinformatics packages such as Mega for the construction of phylogenetic trees (28), Structure for the analysis of population structure (29) or Tassel to perform genome-wide association studies (30). We also implemented options to align reads and call variants in parallel for efficient processing of relatively large numbers of samples. We tested each of these components under a wide range of hardware and operating system environments (Windows, Linux and Mac) to ensure that NGSEP has as much portability as that offered by any Java software package.

We performed a comprehensive validation of the algorithms implemented in NGSEP, compared with other state-of-the-art tools for discovery and genotyping of SNVs, small and large indels and CNVs (Table 1). We reanalyzed a recently published data set of two haploid yeast parents and one pool of *F*_1_ segregants, which we used as a simulated diploid child to test the accuracy of both homozygous and heterozygous genotype calls (4). We also reanalyzed benchmark data sets for the human individual NA12878, who belongs to the population of Utah residents with ancestry from Northern and Western Europe (CEU). We analyzed two samples at different coverage levels released by the 1000 Genomes Consortium. Finally, we performed whole-genome resequencing of rice cultivar IR8, the semi-dwarf variety that significantly increased rice yield in Asia and played an important role in the rice green revolution (31,32).

**Table 1.** Data sets used for testing and comparison of NGSEP with other algorithms for SNV and CNV detection and genotyping

Data set (coverage)	NGSEP		CNVNator		BreakDancer		GATK^b^		SNVer		SAMtools		VarScan 2	
	RT^a^	RAM^a^	RT	RAM	RT	RAM	RT	RAM	RT	RAM	RT	RAM	RT	RAM
Yeast parent CBS6412 (79×)	27 min	1.9 Gb	1 min	1.6 Gb	1 min	10 Mb	1 h 41 min	4.0 Gb	11 min	2.6 Gb	14 min	40 Mb	38 min	5.8 Gb
Yeast parent ER7A (96×)	30 min	2 Gb	1 min	1.6 Gb	1 min	10 Mb	1 h 58 min	4.1 Gb	14 min	2.7 Gb	17 min	40 Mb	36 min	5.8 Gb
Yeast pool (34×)	21 min	1.6 Gb	1 min	1.6 Gb	1 min	15 Mb	1 h 35 min	3.4 Gb	7 min	2.7 Gb	6 min	35 Mb	26 min	5.8 Gb
Rice IR8 (26×)	2 h 38 min	3 Gb	25 min	2.3 Gb	2 h 23 min	2.8 Gb	11 h 21 min	5.8 Gb	–	–	2 h 43 min	100 Mb	–	–
Human chr1 (64×)	1 h 44 min	2.7 Gb	19 min	2.1 Gb	15 min	400 Mb	1 h 37 min	200 Mb	1 h 51 min	2.8 Gb	1 h 28 min	300 Mb	–	–
Human (5×)	9 h 25 min	7.5 Gb	3 h 46 min	7.4 Gb	24 min	500 Mb	8 h 20 min	600 Mb	–	–	5 h 23 min	300 Mb	–	–

^a^ RT: runtime; RAM: random access memory.

^b^ The GATK pipeline was executed as suggested in (9) for the yeast and rice samples. For the human samples only the Unified Genotyper was executed.

SAMtools was the most efficient tool in both time and memory. The GATK pipeline is the most expensive in resource consumption, specifically during polymerase chain reaction (PCR) duplicate identification and quality score recalibration. These two steps take more than three times the time and memory used by the genotyper itself. Because the group of the 1000 Genomes Project already performed duplicate identification and quality score recalibration for the human samples, we ran only the genotyper, obtaining runtimes and memory consumptions similar to those obtained with SAMtools. VarScan 2 was the second most expensive in resource consumption, mainly owing to the perl script for filtering introduced in (13). Because executing this filtering also requires >100 GB of temporary disk space even for the rice sample, we tried only VarScan 2 in the yeast samples. NGSEP uses more time and memory than SAMtools and SNVer owing to the extra steps needed to perform local realignment around indels and to call CNVs and large indels. However, in comparison with the sum of runtimes and maximum memory of CNVnator, SAMtools and BreakDancer, NGSEP showed comparable efficiency for the yeast samples and better overall efficiency for the rice and human samples.

### SNV detection accuracy

**Figure 2.**
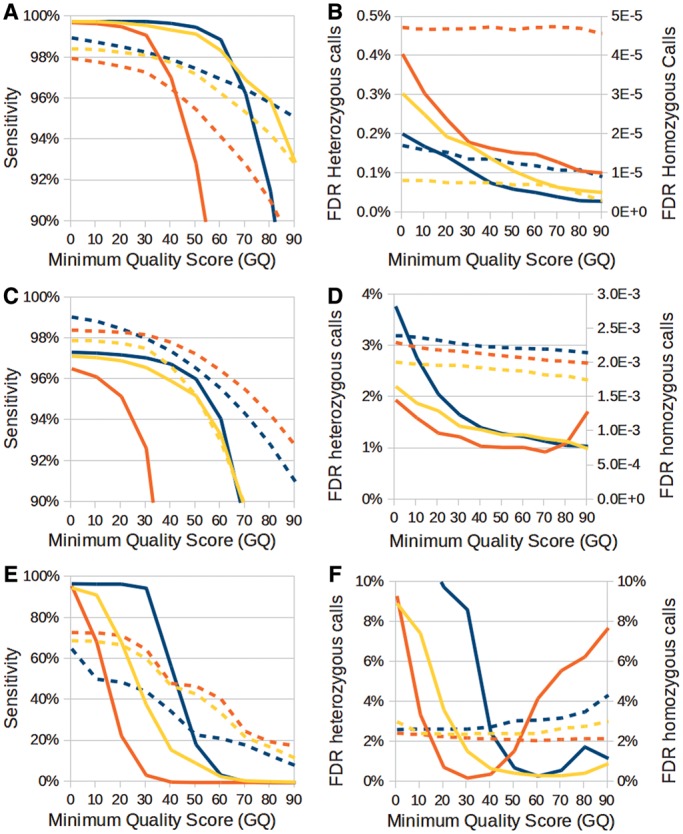
Sensitivity (left panels) and FDR (right panels) for genotyping of SNVs using NGSEP (blue), GATK (red) and SAMtools (yellow) as a function of the minimum quality score on the following benchmark data sets: (**A** and **B**) yeast unselected pool, (**C** and **D**) high-coverage human sample NA12878 and (**E** and **F**) low-coverage human sample NA12878. Continuous lines represent homozygous genotype calls, and broken lines represent heterozygous genotype calls.

We built a gold-standard set of genotypes for the unselected pool of yeast segregants and we calculated the sensitivity and FDR for each tool under different quality filters (see ‘Materials and Methods’ section for details). Figure 2A shows that NGSEP achieved the best sensitivity at the minimum quality threshold below 60 for calling both heterozygous and homozygous SNVs, compared with GATK, and SAMtools. The three methods reported sensitivity >96% even at the minimum quality threshold of 40. The most visible difference among methods is the rapid loss of sensitivity for homozygous SNVs predicted using GATK. For heterozygous genotypes, NGSEP reported between 1 and 6% more sensitivity than GATK and between 0.5 and 2% more sensitivity than SAMtools. The FDR of NGSEP, GATK and SAMtools was always <1% for both homozygous and heterozygous calls (Figure 2B). NGSEP achieved the lowest FDR for homozygous calls at the expense of reporting a larger FDR for heterozygous calls compared with SAMtools. SNVer and VarScan 2 had an inferior performance, especially for heterozygous sites (Supplementary Figure S3). For VarScan, we compared the results before and after applying the filtering step proposed in (13). SAMtools achieved the lowest FDR for heterozygous calls at the expense of reduced sensitivity and higher FDR for homozygous calls. A standard ROC analysis contrasting sensitivity and specificity rates SAMtools as the best package for the discovery of heterozygous genotypes and NGSEP as the best for homozygous genotypes in this data set (Supplementary Figure S4A and B).

We investigated the effect of some of the factors known to affect the SNV genotyping quality. One well-known source of erroneous genotype calls is the confounding effect of PCR amplification artifacts. This problem is tackled in the GATK pipeline with the MarkDup tool of Picard (http://picard.sourceforge.net/index.shtml). However, we found that this tool does not handle well reads with multiple alignments. In NGSEP, we decided to account for amplification artifacts within the algorithm by setting a maximum on the number of reads allowed to start at the same genomic location. We observed a reduction in FDR for heterozygous calls as this parameter becomes smaller at the expense of reduced sensitivity and increased FDR for homozygous calls (Supplementary Figure S5). Another source of genotyping errors usually comes from sequencing errors with high base quality scores. The GATK pipeline includes a module to recalibrate these erroneous quality scores. However, this module consumes more than twice the computing time and memory as the genotyper itself. In NGSEP, we alleviated the problem of miscallibrated quality scores by setting up a parameter to specify the maximum allowed value of a base quality score. Larger scores are equalized to this threshold. Again, we observed a reduction in FDR for heterozygous calls at the expense of sensitivity as we reduced the value of this parameter (Supplementary Figure S6). For maximum values <20, the loss of sensitivity became more important than the reduction in FDR. Finally, we analyzed the effect of changing the tool used to map reads back to the reference. We mapped the reads to both BWA and Bowtie 2 and we ran NGSEP separately on each set of alignments. Results with both tools were almost equal in sensitivity. In the default mode, Bowtie 2 achieved lower FDR for homozygous calls than BWA at the expense of a higher FDR for heterozygous calls (Supplementary Figure S7). We also compared the behavior of Bowtie 2 when it was asked to retain up to three alignments per read (k parameter equals 3) because, as shown below, this parameter has a large influence on the detection of CNVs. We implemented an option in NGSEP to consider secondary alignments for SNV detection and we observed that when this option was activated a modest increase in sensitivity was observed, at the expense of an increase of ∼0.5% in the FDR for heterozygous calls. We finally verified that default NGSEP parameters produced nearly the same results for alignments obtained with Bowtie 2 in the default mode and in the *K* = 3 mode.

For the human data sets, we defined as a gold standard the set of calls available in the Hapmap database for the individual NA12878 (33). Figure 2C shows that, in this case, GATK was slightly better than NGSEP in sensitivity for heterozygous sites at a minimum quality score above 30. However, for homozygous sites, the same loss of sensitivity observed in the yeast sample was observed in this sample. The FDRs (Figure 2D) were generally higher than the ones observed in the yeast sample but they remained low. SAMtools was again the best method to control FDR in heterozygous calls at the expense of sensitivity, followed in this case by GATK (Figure 2D). Comparison based on ROC curves shows that SAMtools still has better compromise between sensitivity and specificity than NGSEP and GATK for heterozygous sites (Supplementary Figure S4C). For homozygous sites, NGSEP still shows the best compromise but the difference with SAMtools and GATK became minimal (Supplementary Figure S4D). However, it is worth noting that the compromise between sensitivity and specificity obtained by NGSEP and SAMtools at minimum quality 40 (ticker datapoints) can be obtained only with GATK at minimum quality below 20.

We also compared the results of the different algorithms in the newer low-coverage data set for NA12878. Because the coverage for this data set is only 5×, it became more difficult for all algorithms to predict the right genotype, especially at heterozygous sites (Figure 2E). Again, GATK had the best sensitivity for heterozygous sites but the worst for homozygous variant sites. The FDR remained low for heterozygous calls but it increased for homozygous variant calls mainly because a large number of heterozygous sites were incorrectly called as homozygous variants (Figure 2F). Comparison based on ROC curves suggests a small advantage of GATK for heterozygous sites (Supplementary Figure S4E). For homozygous sites all methods show similar compromise between sensitivity and specificity (Supplementary Figure S4F). However, in this case the lower coverage forces both SAMtools and GATK to reduce their minimum quality threshold below 20 to obtain the same compromise obtained by NGSEP at minimum quality 40. Finally, it is worth noting that, for minimum quality scores 40 and 50, even at a small coverage of 5×, the three methods achieved FDRs <3% for both homozygous and heterozygous genotype calls.

### Detection accuracy for small indels

**Figure 3.**
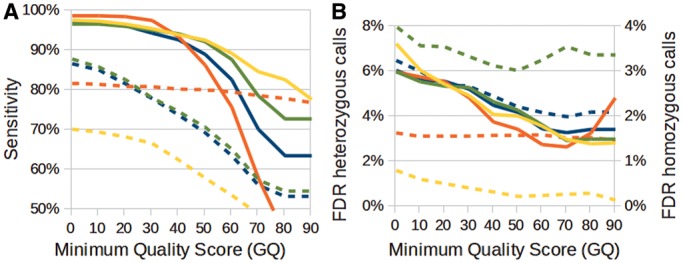
(**A**) Sensitivity and (**B**) FDR for genotyping of small indels produced by NGSEP (blue), GATK (red) and SAMtools (yellow) using reads aligned with BWA, and NGSEP (green) using reads aligned with Bowtie 2 on the yeast unselected pool as a function of the minimum quality score. Continuous lines represent homozygous genotype calls, and broken lines represent heterozygous genotype calls.

To validate the accuracy of small indel detection, we defined a gold-standard data set for the unselected sample using the same approach we used to validate SNV detection accuracy. In general, the percentage of agreement between NGSEP and GATK was only ∼80%, which is much lower than the percentage obtained for SNVs (99%). SAMtools was the most sensitive for homozygous indels at the expense of having the worst sensitivity for heterozygous indels and the largest FDR for homozygous genotype calls (Figure 3 and Supplementary Figure SS8). GATK was the best algorithm for genotyping of heterozygous indels, and the drop in sensitivity observed in the validation of homozygous SNVs was less pronounced in this case. Although NGSEP seemed to have the largest error rate for heterozygous indels, it is worth mentioning that for quality scores >40, the absolute difference between NGSEP and GATK in type 1 errors was just 8 and this difference disappears for a minimum quality score of 60. We also compared the accuracy of NGSEP using BWA and Bowtie 2 alignments and we found that Bowtie 2 is able to identify more homozygous indels at the expense of increased FDR for heterozygous calls.

### CNV detection accuracy

**Figure 4.**
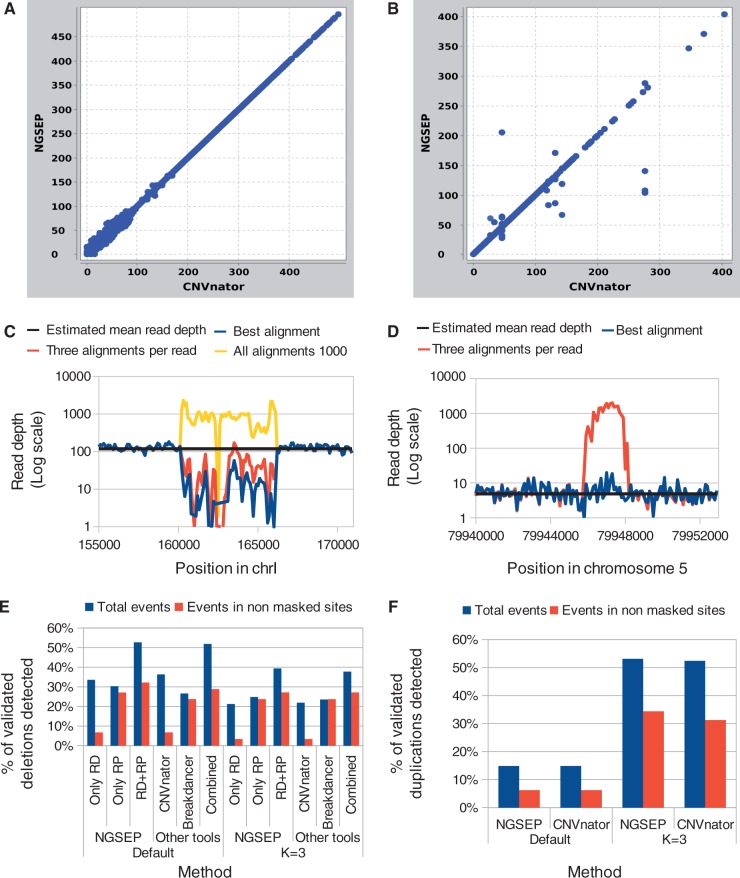
Quality assessment of the implementation of the CNVNator algorithm in NGSEP. Given the same GC-corrected intensities, the same genome size and the same RD distribution parameters, both implementations produce nearly the same (**A**) partition and (**B**) RD levels. Examples of repetitive regions in (**C**) the yeast parent ER7A and (**D**) the low-coverage human sample show how RD varies depending on the number of alignments counted for each read (blue: only the best alignment of each read counted; red: up to three alignments counted; yellow: all alignments found with Bowtie 2 with the -a option counted). Sensitivity of NGSEP, CNVnator and BreakDancer to identify (**E**) deletions and (**F**) duplications validated by Mills and collaborators (22) using reads from the low-coverage data set for NA12878. Default and K = 3 modes of Bowtie 2 are compared.

We tested our implementation of the CNVnator algorithm on the different data sets to make sure that both implementations provide similar results. In general, the percentage of agreement for the yeast samples was >90% and rose to 98% for the human samples, mainly because the original implementation of CNVnator has parameters such as the size of the genome fixed for processing of human samples. Conversely, in NGSEP, the genome size is determined from the reference file or can even be set up as a parameter. The other important difference between the two implementations is that, to calculate averages and standard deviations for both GC-correction and determination of the average RD, we consider only bins without reads with multiple alignments. This allows us to process samples in which multiple alignments are recorded. We verified that, given the same read intensities, the same genome size and the same predicted average and standard deviation on the RD, our implementation produces the same partition and almost the same CNV calls (Figures 4A and B).

To compare the effects of different read alignment strategies on CNV detection, we performed three independent runs of Bowtie 2 for each of the yeast data sets keeping (i) the best alignment for each read (default), (ii) up to three alignments per read (-k option equal to 3) and (iii) all possible alignments for each read (-a option) (see ‘Materials and Methods’ section for details). We compared the CNV calls reported in the haploid parents CBS6412 and ER7A with a database of 700 known repeat elements available in the *Saccharomyces cerevisiae* database (http://www.yeastgenome.org/download-data/curation). As expected, using the third approach, 33 and 47% of the repeats were identified by NGSEP on the alignments for CBS6412 and ER7A, respectively. Conversely, the first approach reported only between 0.5 and 2% of the repeats. Interestingly, between 40 and 48% of the repetitive regions were called deletions using the first approach. Using the intermediate approach NGSEP called between 5 and 10% of the repetitive regions as duplications and between 14 and 22% as deletions. As an example to explain this behavior, Figure 4C shows how read intensities differ for a long terminal repeat (LTR) located on chromosome I. While the third approach was able to retain all reads that belong to any of the copies of this element present in the genome, the other two approaches retained only a small percentage, which in this case was much smaller than the average RD for unique regions. This resulting pattern should produce a false positive deletion call for this region using any CNV detection algorithm based on RD.

The same confounding effect of repetitive regions could be observed in the human samples. From the gold-standard data set of 642 deletions and 271 duplications developed by Mills and coworkers (22), only 32 duplications and 59 deletions do not overlap annotated repetitive elements. Because in most of the previous studies only the best alignment is kept for each read, most of the validated duplications are classified as ‘not accessible by RD algorithms’ (14,34). Figure 4D shows an example of a validated duplication that becomes accessible in the low-coverage data set after mapping reads with Bowtie 2 and keeping three alignments for each read. Although this region is not masked as a repeat in the current reference genome, it is annotated as a nuclear mitochondrial sequence (NUMT region) (35). We found two almost perfect copies of this region on chromosomes 11 and on the mitochondrial chromosome, respectively.

Figure 4E and F show the percentage of validated deletions and duplications for the human sample NA12878 identified by NGSEP, CNVnator and BreakDancer in the low-coverage data set. For NGSEP, we compared the detection power of the RD and the RP algorithms, and we also included the results obtained combining both approaches. As expected, results with the RD approach implemented in NGSEP were similar to the results obtained using the original implementation of CNVnator. Keeping three alignments per read reduced the percentage of detected deletions but increased by >30% the percentage of detected duplications. Combining the RD and RP approaches increased by >10% the percentage of detected deletions compared with the results using only the RD approach. The combination of RD and RP algorithms of NGSEP showed a slightly better sensitivity than the combination of the results produced by CNVnator and BreakDancer.

Because the identification of repetitive regions is important for both SNV and CNV detection, we implemented an option in the variants detector to identify repetitive regions in the genome based on reads with multiple alignments. This module traverses the read alignments sorted by genomic location and clusters overlapping multiple alignments into candidate repetitive regions. Genomic regions in which <20% of the alignments are unique are called repetitive. We were able to detect >90% of the 512 272 base pairs annotated as repetitive in the yeast genome. Even using the low-coverage data set for NA12878 we recovered 16% of the 1.7 Gb annotated as repetitive elements in the human genome. NGSEP integrates predicted repeat elements, CNVs and large indels obtained by each of the algorithms described above into a single GFF file. This GFF file can be directly uploaded to a genome browser for detailed visualization of structural variants in any genomic region of interest.

### Resequencing of the green revolution rice cultivar IR8

We performed whole-genome resequencing of rice cultivar IR8, the semi-dwarf indica variety developed by the International Rice Research Institute that played an important role during the green revolution. We mapped reads to the current reference genome (36) using Bowtie 2 with the option k = 3 to keep up to three alignments per read and then we performed discovery of genomic variants with NGSEP. Looking at the sequencing error rate predicted by the quality statistics produced with NGSEP (Supplementary Figure S9), we chose to ignore the last four base pairs of each read to detect of SNVs and small indels. We also set the prior heterozygosity rate to 0.0001 to take into account that IR8 went through several generations of inbreeding. Finally, we set the maximum base quality score to 30, the maximum number of alignments with the same start to 2 and the minimum genotype quality to 40. NGSEP detected 59 322 repeat regions, 13 427 duplications, 18 362 large deletions and 5120 large insertions spanning 125.4, 148.07, 39.53 and 1.54 Mb, respectively. In all, 63.76% of the predicted duplications and 43.89% of the predicted deletions were located in sites identified as repetitive. The total genomic length of regions identified as repetitive or with abnormal copy number was 200.68 Mb. To check the consistency of our findings with previous work, we calculated the regions masked as repetitive in the version of the reference genome available in phytozome (www.phytozome.net) (37). From 128.44 Mb masked as repetitive in this version of the reference, NGSEP identified 105.84 Mb (82.4%) as repeats or regions with abnormal copy number.

NGSEP found 4 266 169 SNVs and 315 834 small indels, from which 1 657 880 (38.86%) and 58 494 (18.52%) were heterozygous. At first glance, this result seemed to be inconsistent with the expected loss of heterozygosity produced by the successive generations of inbreeding performed to develop IR8. However, we could verify that a large percentage of these heterozygous calls really represented differences between copies of repetitive elements. The percentage of heterozygous calls decreases to 10.35% if we filter out variants falling into repetitive regions and CNVs identified by NGSEP (Supplementary Table S2). Filtering SNVs in the currently available masked regions reduced the same percentage to only 20.21%. A similar behavior was observed in the percentage of heterozygous indels and in the percentage of heterozygous variants in coding regions. Moreover, we observed a reduction in the percentage of nonsense SNVs and frameshift indels. A possible reason for this phenomenon is that selective pressure does not need to be as strong in genes with multiple copies as it is for single-copy genes because a damaging mutation in one copy of a paralog gene does not produce complete loss of function. In fact, nonsynonymous mutations between paralog copies of genes could confer beneficial neofunctionalization (38).

**Figure 5.**
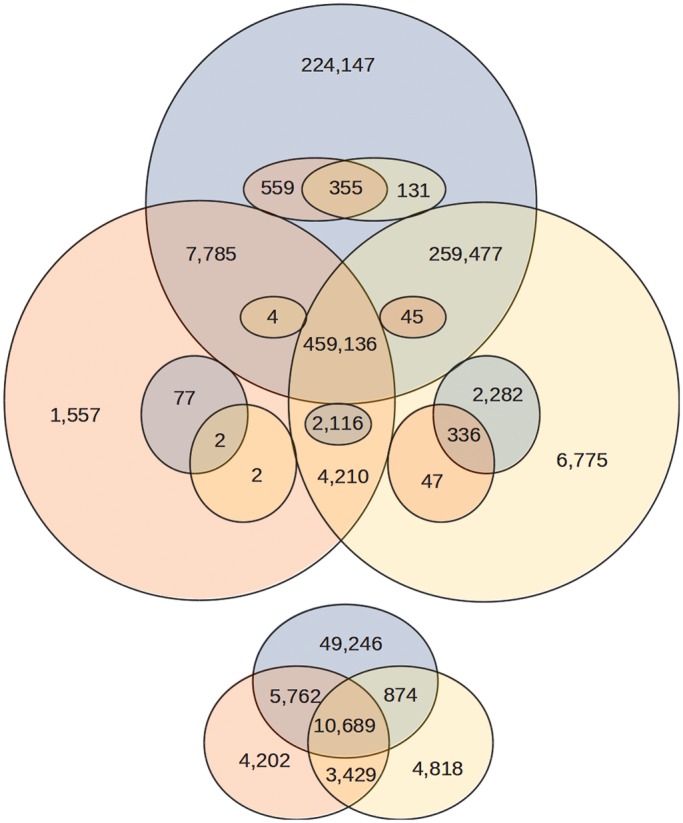
Venn diagrams comparing the variants discovered by NGSEP (blue), GATK (red) and SAMtools (yellow) on the data set of reads obtained after sequencing rice cultivar IR8. The upper diagram compares homozygous nonreference calls among the three methods. Smaller circles within each category represent sites that were called heterozygous by at least one method and homozygous nonreference by at least another method (for example, 2116 variants called homozygous nonreference by GATK and SAMtools were called heterozygous by NGSEP). The smaller diagram at the bottom compares heterozygous calls that were not called homozygous nonreference by any of the three methods.

For comparison, we also ran the GATK pipeline and SAMtools, both based on BWA alignments. NGSEP reported 1 023 023 SNVs after the most stringent filtering of repetitive regions, compared with 500 313 SNVs reported by GATK and 754 728 SNVs reported by SAMtools using the same filters. NGSEP also reported >2-fold and 1.25-fold more indels and variants in coding regions than GATK and SAMtools, respectively (Supplementary Table S2). Unfortunately, in this case, we do not have gold-standard genotype calls to make a comprehensive assessment of the sensitivity and specificity achieved by the different algorithms. As indirect measures of quality, we calculated the percentage of nonsynonymous SNVs, the percentage of nonsense SNVs and the percentage of frameshift indels. The three methods reported similar values for these measures, which allows us to speculate that the three methods have similar overall specificity. SAMtools reported consistently lower percentages of heterozygous variants, which, as observed in the experiments on yeast and human samples, is likely to be due to better specificity for heterozygous calls. We also built a Venn diagram to assess the agreement among the three methods (Figure 5). More than half of the variants reported by NGSEP and not reported by GATK were reported by SAMtools, which provides increased confidence in the genotype calls reported by NGSEP. To assess whether GATK and SAMtools call a homozygous reference genotype or left uncalled the variants called only by NGSEP, we compared genotype calls reported by GATK and SAMtools at lower qualities and we found that both tools were able to call up to 92% of the variants reported by NGSEP if their minimum quality was lowered to zero (Supplementary Figure S10). Finally, we queried the 273 393 SNVs only identified by NGSEP in a data set of 60 sequenced rice varieties and we found that, from 208 923 SNVs for which at least 30 samples were genotyped, 183 256 (87.71%) have minor allele frequencies >0.05 and only 14 180 (6.79%) are unique for IR8 (manuscript in preparation).

The genetic cause for the reduced height of IR8 is a deletion of 382 bp spanning exons 1 and 2 of the *SD1* gene (32). We verified that the RP algorithm implemented in NGSEP was able to detect this deletion. We uploaded the GFF produced by NGSEP to the genome browser available on the rice genome project Web site (http://rice.plantbiology.msu.edu) and we could observe that the deletion predicted by NGSEP covers the validated deletion with almost perfect base-pair resolution (Supplementary Figure S11).

## DISCUSSION

Despite the exponential reduction in sequencing cost produced by the technological advances achieved in the past 10 years, analysis of HTS data remains a complex process that requires large investments in computational capacity and personnel to translate sequencing data into valuable information such as genes related to traits or markers for selection. NGSEP is the product of an effort to contribute with the evolution of software tools to facilitate the analysis of HTS data. NGSEP provides an intuitive framework in which scientists can analyze their sequenced samples and obtain comprehensive data sets of genomic variants. We used data sets of different species and different natures to show that NGSEP has high accuracy, efficiency and applicability. The use of standard file formats for both receiving input data and producing variant calls enables an easy integration with different visualization and analysis tools.

We built on the experience obtained in our previous work to tackle common issues affecting detection and genotyping of SNVs, such as ambiguously called reads, miscalibrated quality scores, PCR amplification artifacts and misalignment around small indels. Because we followed different strategies to solve these problems compared with those adopted in the GATK pipeline, we demonstrated that our solutions are more efficient in the consumption of computational resources and in general report improved sensitivity for similar specificity.

To comprehensively assess the different algorithms for detecting and genotyping SNVs and small indels, we constructed a gold-standard data set of genotype calls for an *F*_1_ pool of yeast segregants covering >60% of the yeast genome. In contrast with previous studies in which only general measures such as transition/transversion ratio or degree of sharing among algorithms were used as indirect indicators of quality (5,11), this gold-standard allowed us to make precise estimations of sensitivity and specificity for the most widely used software tools under different quality filters. Our comparisons indicate that NGSEP, SAMtools and GATK are the most competitive alternatives. However, the results presented here are only a snapshot of the current state, which is likely to change with the evolution of current alternatives and the development of new algorithms. In this context, the availability of a genome-wide gold-standard data set is a valuable resource for performing continuous comparisons of current and novel algorithms and for promoting the future development of accurate genotyping methods.

As recent studies have pointed out, accurate discovery of CNVs from HTS data is an extremely challenging task and the current performance of the available algorithms is far from satisfactory. For NGSEP, we decided to reimplement the recently developed algorithm CNVnator, which is based on a robust statistical analysis of the RD signal. Because for this kind of approach the management of reads with multiple alignments plays a critical role, we compared different alignment strategies implemented in BWA and Bowtie 2 mapping tools. In general, we found that keeping multiple alignments increases sensitivity to identify duplications. Because proper identification of repetitive elements is critical to make a good interpretation of both SNVs and CNVs detected by any bioinformatics pipeline, we also included a module in the variants detector to explicitly find repetitive regions in the genome based on the information provided by the reads with multiple alignments. Finally, we combined CNVnator with a RP algorithm and we verified that combining these two approaches increases sensitivity for detecting large deletions. Sequencing of rice cultivar IR8 shows that NGSEP provides a more accurate separation between heterozygous variants and differences between copies of repetitive elements compared with other commonly used pipelines. The RP algorithm implemented in NGSEP was also able to identify the large deletion in the *SD1* gene, which shows that NGSEP can be helpful in identifying variants conferring important phenotypic effects.

We believe that the main advantage of NGSEP is the out-of-the-box integration of accurate algorithms to discover, genotype, combine, annotate and filter genomic variants, which facilitates the analysis and understanding of the final results. Although it could be argued that this is already offered by web portal solutions such as Galaxy (39), in practice the initial set up, integration of pipelines and maintenance of local Galaxy installations still require a significant amount of technical support. NGSEP offers an alternative in which scientists can discover, genotype and perform downstream analysis of genomic variants on their local computers, requiring only as much support as that needed by a standard desktop application. Moreover, the level of integration among algorithms achieved by NGSEP allows researchers to summarize diversity, copy number and functional information into a single VCF file. Given the heterogeneity in programming languages, quality and input and output formats observed in current bioinformatic solutions, achieving the same integration with a combination of existing tools still requires significant programming and scripting efforts. However, NGSEP is not meant to replace cluster solutions, which are more suitable for large projects involving analysis over hundreds or even thousands of samples. For these kinds of projects, we offer the main functionalities of NGSEP through a command line interface that allows parallelization in a cluster environment. The main functions are also described in XML scripts, allowing groups with local galaxy installations already in place to use NGSEP, combine it with other tools, and benefit from the flexibility offered by web portals such as Galaxy.

In our local experience, we have successfully used NGSEP to process 60 whole-genome sequencing samples of rice and close to 200 restriction site associated DNA (RAD) sequencing samples of cassava using our local computational cluster. We have also been able to analyze a cassava GBS population of 77 samples within 2 days through the graphical interface in a normal desktop (unpublished data). We believe that NGSEP will become a great alternative for a broad range of scientists to analyze HTS data in their current and future projects.

## ACCESSION NUMBERS

Sequencing reads for the yeast samples analyzed in this work are available on the NCBI short read archive database (http://www.ncbi.nlm.nih.gov/sra) with accession number SRA054394. Sequencing reads for rice cultivar IR8 are also available at SRA with accession number SRR869317.

## SUPPLEMENTARY DATA

Supplementary Data are available at NAR Online.

## FUNDING

International Center for Tropical Agriculture (CIAT); the National Science Foundation (NSF) [0965420 to J.T.]; the Agentschap voor Innovatie door Wetenschap en Technologie (IWT) Flanders [SBO IWT50148, IWT90043 to J.M.T.]; the European Commission (EC) 7th Framework program (NEMO project) (to J.M.T.); the Katholieke Universiteit Leuven (KU Leuven) Industrieel Onderzoeksfonds (IOF) Knowledge platform IKP/10/002 [ZKC1836 to J.M.T.], Bijzonder Onderzoeksfonds (BOF) Program financing (project NATAR) (to J.M.T.); the European Research Council (ERC) [Young Investigator grant 241426 to K.J.V.]; the Vlaams Instituut voor Biotechnologie (VIB); the Fonds Wetenschappelijk Onderzoek (FWO) Vlaanderen (to K.J.V.); the Odysseus program (to K.J.V.); and the European Molecular Biology Organization (EMBO) International Youth Initiative Program (YIP) (to K.J.V.). Funding for open access charge: International Center for Tropical Agriculture (CIAT) Core Funding.

*Conflict of interest statement*. None declared.

## Supplementary Material

Supplementary Data
