# Supplementary material for: An integrated framework for discovery and genotyping of genomic variants from high-throughput sequencing experiments
Source: Nucleic Acids Res. 2014 Jan 11;42(6):e44. doi: 10.1093/nar/gkt1381 (PMC3973327; doi:10.1093/nar/gkt1381)
Supplement: Supplementary Data [file supp_42_6_e44__index.html]

An integrated framework for discovery and genotyping of genomic variants from high-throughput sequencing experiments — An integrated framework for discovery and genotyping of genomic variants from high-throughput sequencing experiments — Supplementary Data 

# An integrated framework for discovery and genotyping of genomic variants from high-throughput sequencing experiments

## Supplementary Data

files

**Files in this Data Supplement:**

- Supplementary Data - pdf file
