# Supplementary material for: An integrated framework for discovery and genotyping of genomic variants from high-throughput sequencing experiments
Source: Nucleic Acids Res. 2014 Jan 11;42(6):e44. doi: 10.1093/nar/gkt1381 (PMC3973327; doi:10.1093/nar/gkt1381)
Supplement: Supplementary Data [file supp_gkt1381_nar-03143-met-n-2013-File009.pdf]

Supplementary tables, figures, and scripts for:

An integrated framework for discovery and genotyping of genomic variants from high throughput sequencing experiments

Jorge Duitama[1,*], Juan Camilo Quintero[1], Daniel Felipe Cruz[1], Constanza Quintero[1], Georg Hubmann[2,3], Maria Foulquié-Moreno[2,3], Kevin J. Verstrepen[4,5], Johan M. Thevelein[2,3] and Joe Tohme[1]

[1] Agrobiodiversity research area, International Center for Tropical Agriculture (CIAT), A.A. 6713, Cali, Colombia
[2] Laboratory of Molecular Cell Biology, Institute of Botany and Microbiology, KU Leuven, Kasteelpark Arenberg 31, B-3001 Leuven-Heverlee, Flanders, Belgium.
[3] Department of Molecular Microbiology, VIB, Kasteelpark Arenberg 31, B-3001 Leuven-Heverlee, Flanders, Belgium
[4] VIB Laboratory of Systems Biology, KU Leuven, Gaston Geenslaan 1, B-3001 Leuven-Heverlee, Flanders, Belgium
[5] Laboratory for Genetics and Genomics, Centre of Microbial and Plant Genetics, KU Leuven, Gaston Geenslaan 1, B-3001 Leuven-Heverlee, Flanders, Belgium

Supplementary Table 1. Comparison of functional features among the different software tools included in this study

	Read analysis		Variant detection				Genotype calls postprocessing			Usability	
Tool	Alignment	Sorting	SNVs and Small indels	CNVs	Large indels	Merging samples	Functional annotation	Filtering	Format Conversion	Graphical interface	Galaxy integration
NGSEP	X	X	X	X	X	X	X	X	X	X	X
BWA	X										X
Bowtie 2	X										X
Picard		X									X
GATK			X			X		X	X		X
SAMtools		X	X			X		X			X
VarScan			X			X		X			
SNVer			X							X	
CNVnator				X							
BreakDancer					X						

Supplementary Table 2. SNVs and small indels found by NGSEP, the GATK pipeline, and SAMtools using the IR8 dataset and filtered by different sets of repetitive and abnormal copy number regions. For each count, the percentage of heterozygous variants is shown in parentheses

		SNVs				Indels		
Algorithm	Filter	Total (% het)	Coding (% het)	% Missense	% NonSense	Total (% het)	Coding (% het)	% frameshift
NGSEP	None	4266169 (38.86%)	754755 (52.15%)	52.37%	2.10%	315834 (18.52%)	12410 (29.69%)	64.09%
	Masked sites	1972961 (20.21%)	285756 (25.66%)	58.02%	2.04%	204273 (11.14%)	7728 (19.53%)	61.15%
	Repeats	2197026 (14.69%)	244718 (16.7%)	57.28%	1.87%	247546 (10.19%)	8133 (19.82%)	56.09%
	Repeats + CNVs	1444176 (10.35%)	155275 (9.45%)	56.01%	1.55%	176655 (7.84%)	5366 (16.34%)	51.96%
	Repeats + CNVs + Masked sites	1023023 (6.98%)	136527 (8.98%)	55.36%	1.41%	132089 (5.75%)	4351 (11.61%)	55.39%
GATK	None	1522651 (17.6%)	174290 (35.43%)	57.06%	2.72%	150677 (8.1%)	3479 (24.81%)	73.21%
	Masked sites	868536 (13.54%)	100042 (23.75%)	57.48%	2.10%	97271 (6.79%)	2292 (20.38%)	71.60%
	Repeats	1139454 (9.18%)	102861 (19.6%)	57.53%	2.15%	132846 (4.72%)	2622 (16.02%)	68.46%
	Repeats + CNVs	729461 (5.26%)	60749 (9.37%)	55.63%	1.68%	91590 (2.53%)	1452 (7.23%)	64.39%
	Repeats + CNVs + Masked sites	500313 (5.08%)	54179 (9.49%)	54.89%	1.49%	64171 (2.25%)	1176 (7.74%)	67.18%
SAMtools	None	2127593 (8.65%)	274843 (16.88%)	58.65%	2.51%	221694 (3.76%)	7828 (12.63%)	56.63%
	Masked sites	1244598 (7.63%)	166778 (12.46%)	56.99%	1.91%	148967 (3.38%)	4975 (10.39%)	56.58%
	Repeats	1573630 (5.02%)	167372 (10.79%)	56.99%	1.95%	193457 (2.78%)	6058 (11.08%)	49.32%
	Repeats + CNVs	1064775 (2.45%)	111168 (4.59%)	55.34%	1.58%	140262 (1.88%)	4107 (8.77%)	43.49%
	Repeats + CNVs + Masked sites	754728 (2.69%)	99754 (4.7%)	54.57%	1.39%	103286 (1.64%)	3042 (5.82%)	49.70%

Supplementary Figure 1. Schematic of a generic pipeline for variants discovery from high throughput sequencing data for cases where a reference genome is available

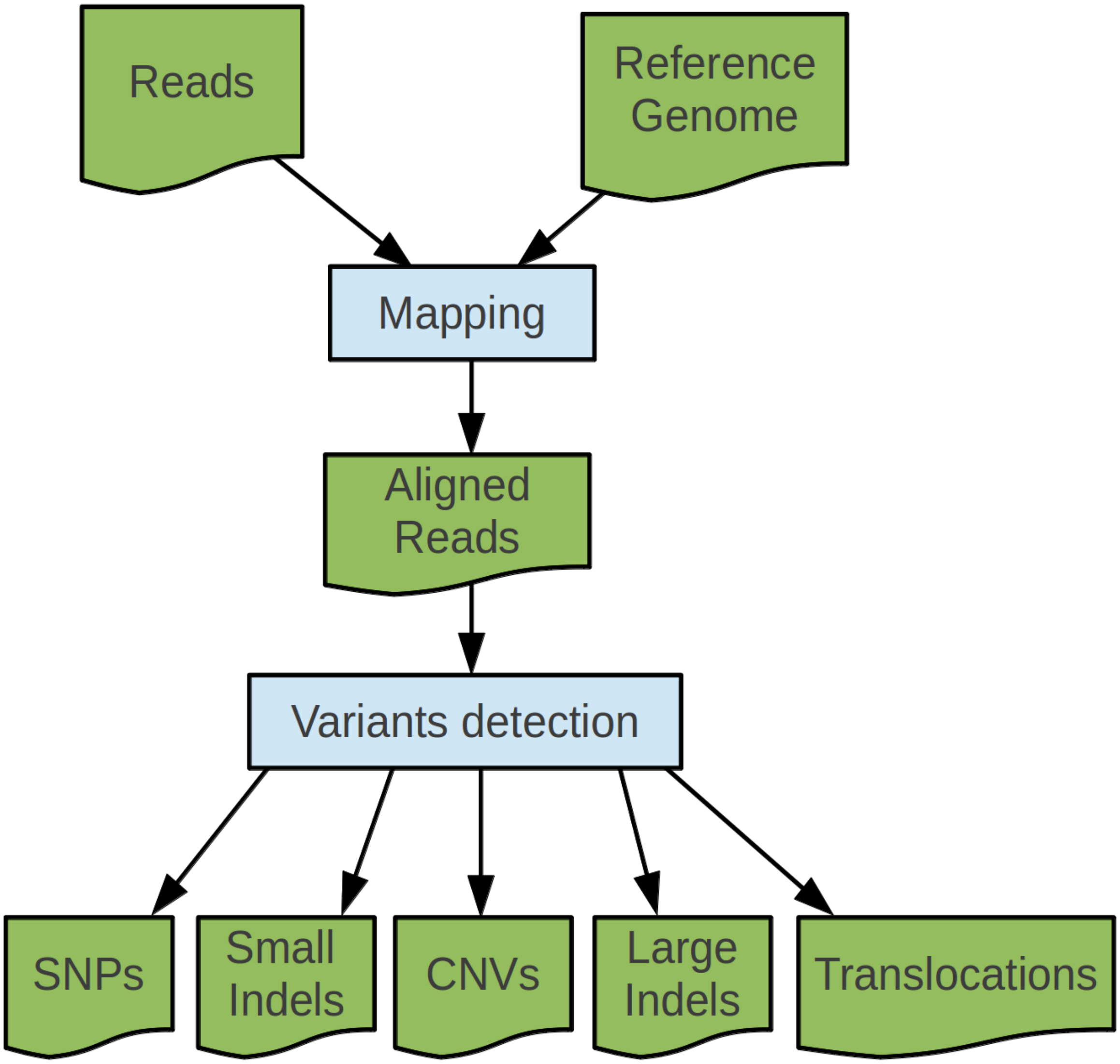

Supplementary Figure 2. (A) Construction of gold standard genotypes for a pool of F1 yeast segregants obtained from independent crosses of two haploid parents. The two parents are sequenced at 80x and high quality genotypes are called for both parents in sites in which only one allele is observed. Only sites in which NGSEP, GATK, SAMtools, and VarScan agree are retained. Sites in which the two parents agree (blue) are predicted to be homozygous in the pool. Sites in which the parents disagree (red for the CBS6412 allele, yellow for the ER7A allele) are predicted to be heterozygous in the pool. (B) The observed proportion of the reference allele in the sites predicted as being heterozygous is consistent with a simulated distribution taking into account genetic drift and variation in read depth.

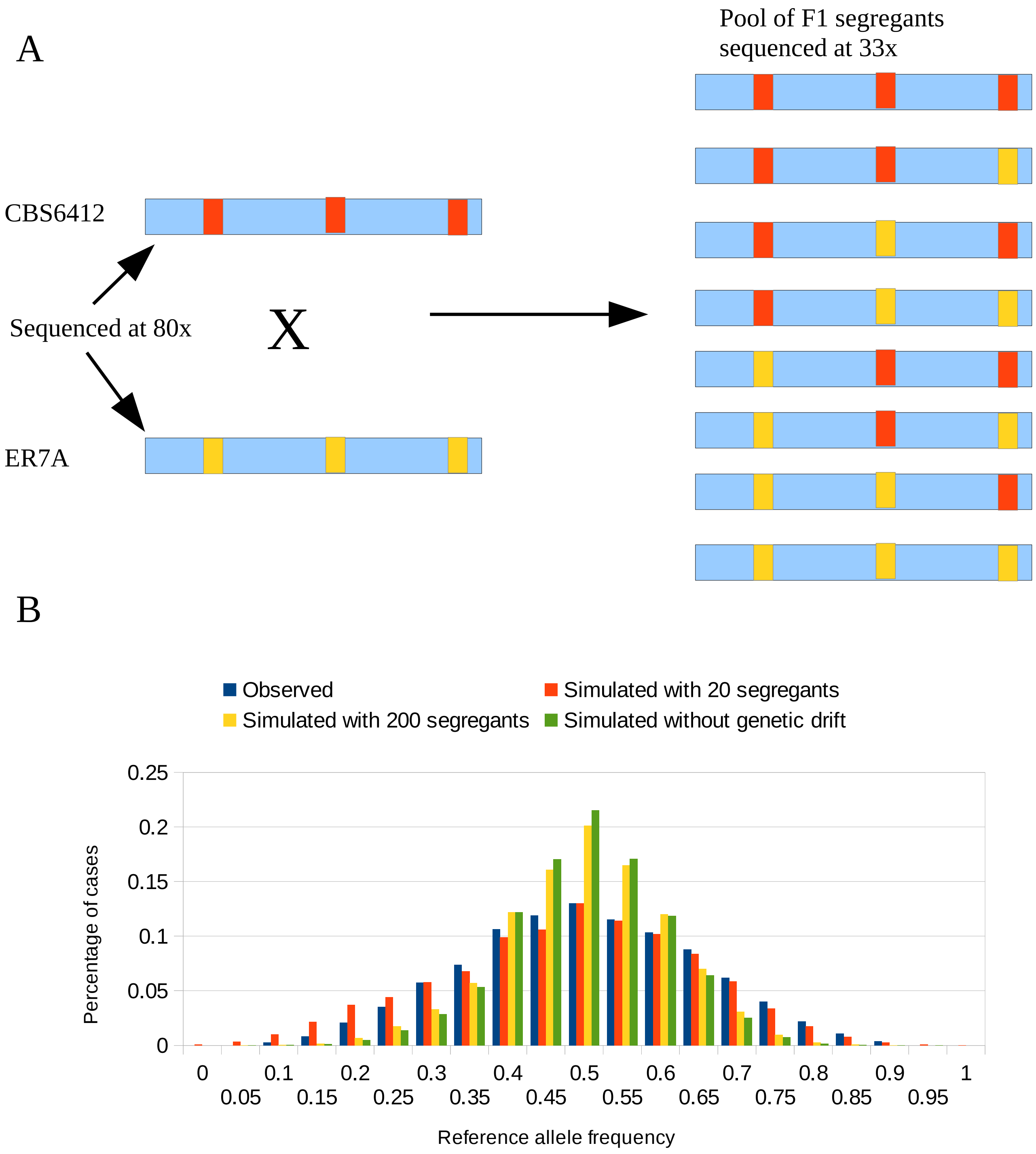

Supplementary Figure 3. Sensitivity and false discovery rate (FDR) obtained by NGSEP (blue), SNVer (red), VarScan (yellow), and VarScan 2 (green). Continuous lines represent homozygous genotype calls and dashed lines represent heterozygous genotype calls.

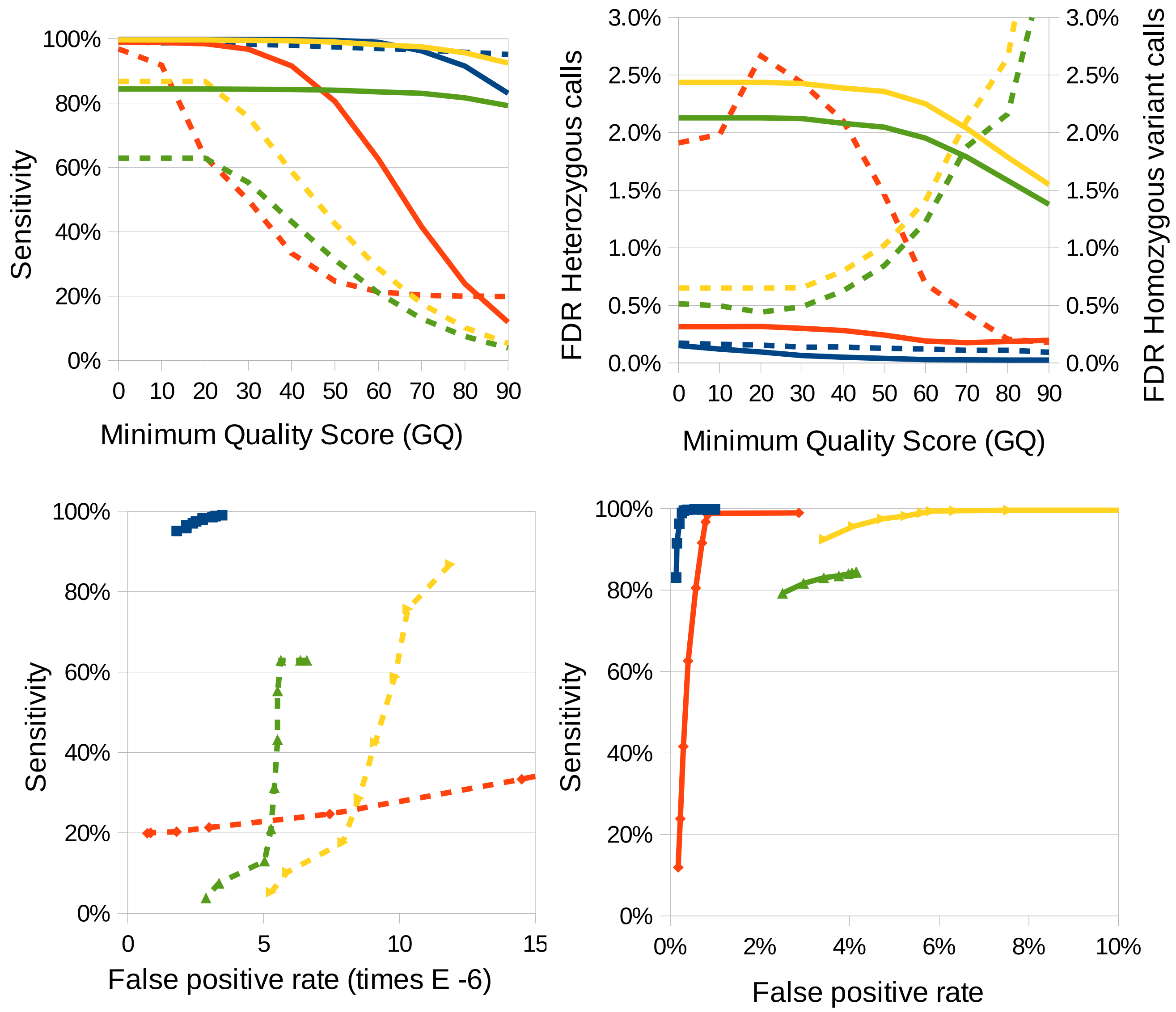

Supplementary Figure 4. ROC curves comparing sensitivity and specificity for heterozygous genotype calls (left panels) and homozygous genotype calls (right panels) produced using NGSEP (blue), GATK (red), and SAMtools (yellow) on (A and B) the yeast unselected pool, (C and D) the high-coverage human sample NA12878, and (E and F) the low-coverage human sample NA12878.

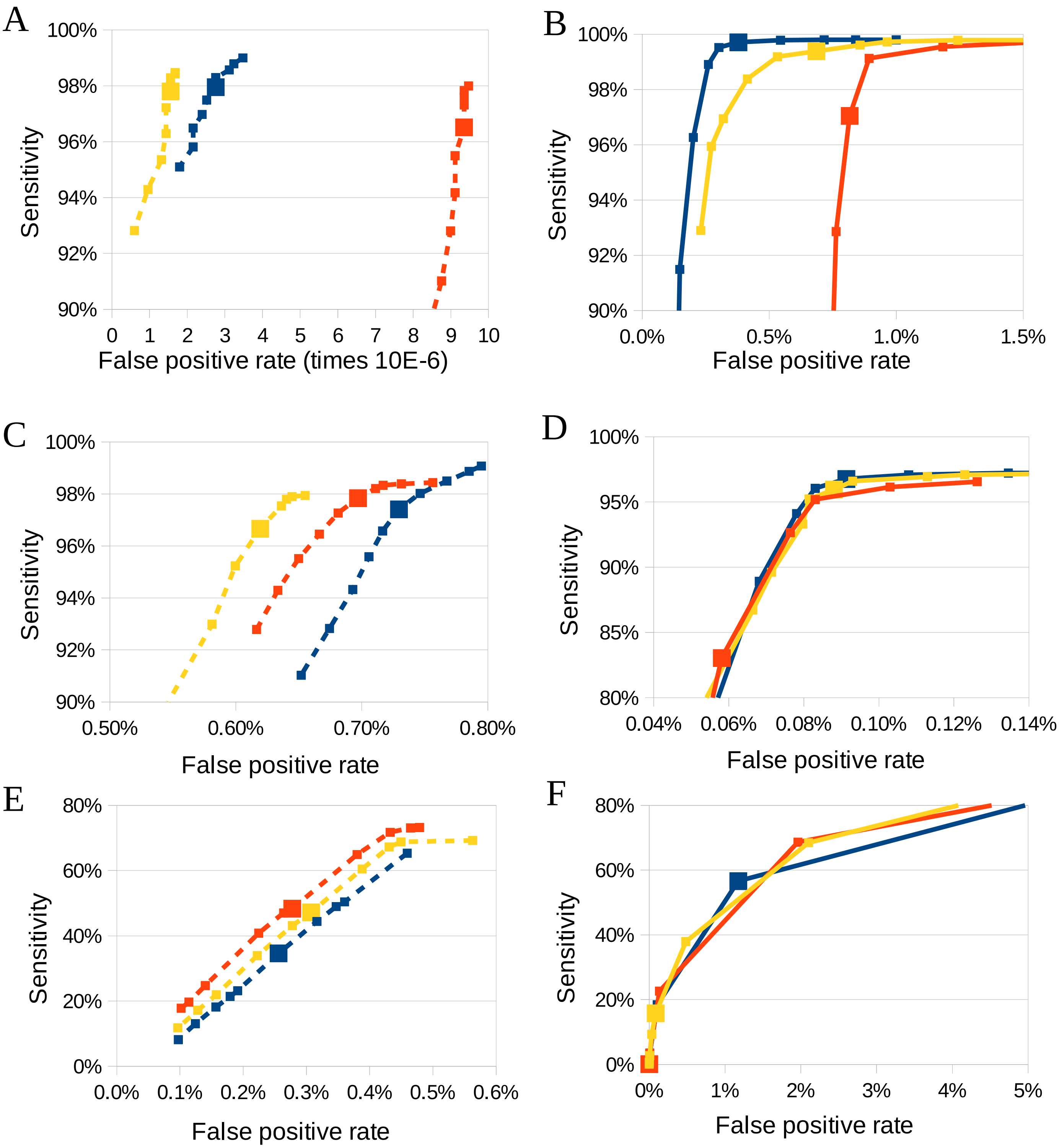

Supplementary Figure 5. Sensitivity and false discovery rate (FDR) obtained by NGSEP setting the number of alignments allowed to start at each genomic site to one (red), two (green), four (yellow) and no limit (blue). Continuous lines represent homozygous genotype calls and dashed lines represent heterozygous genotype calls.

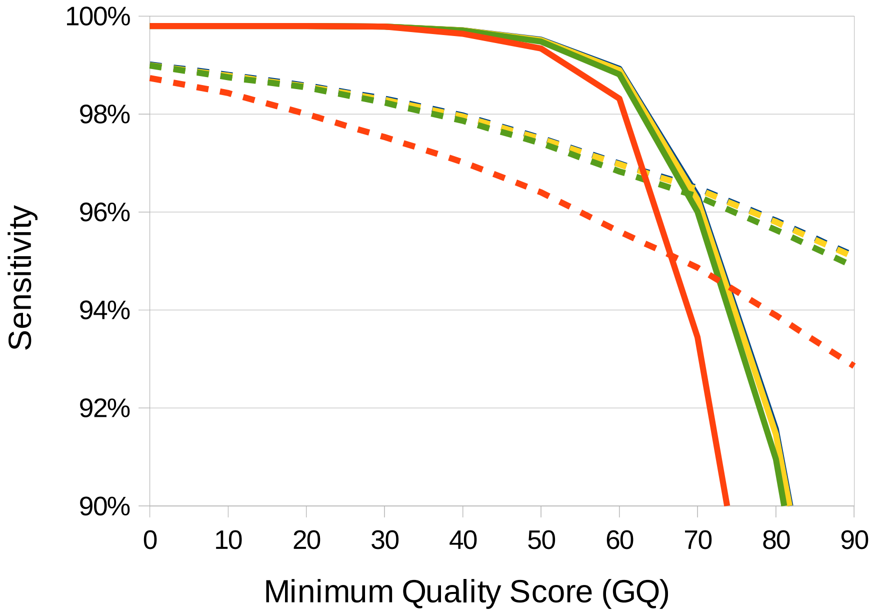

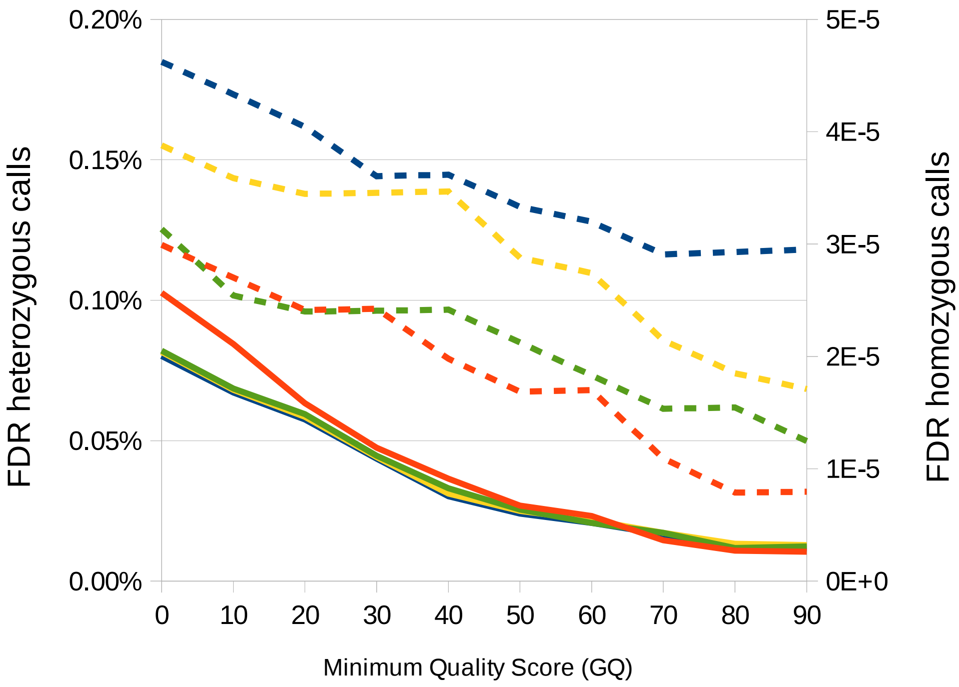

Supplementary figure 6 Sensitivity and false discovery rate (FDR) obtained by NGSEP setting the maximum base quality score to 20 (green), 30 (yellow) and no limit (blue). Continuous lines represent homozygous genotype calls and dashed lines represent heterozygous genotype calls.

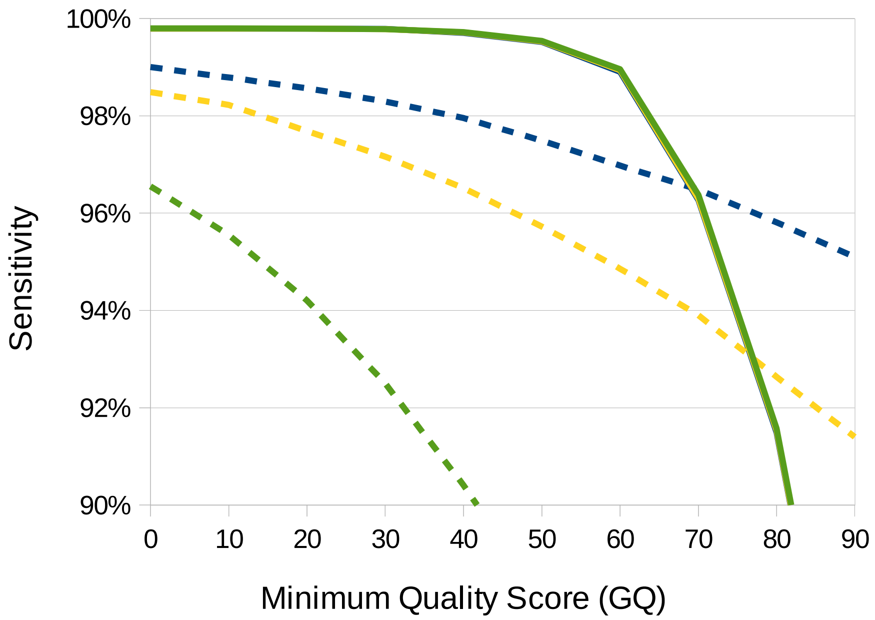

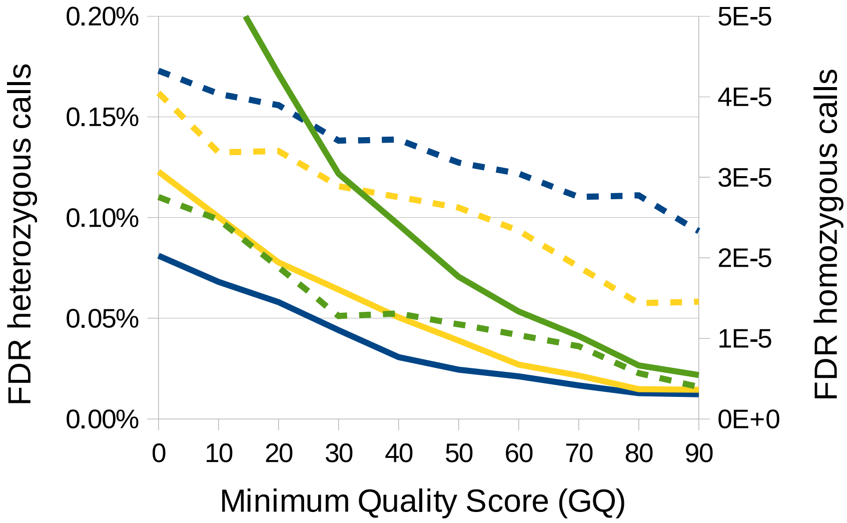

Supplementary figure 7. Sensitivity and false discovery rate (FDR) obtained by NGSEP on alignments obtained with BWA (blue), Bowtie 2 in default mode (red) and Bowtie 2 with the k option equals 3 and activating in NGSEP the option to consider secondary alignments for SNV detection (yellow). Continuous lines represent homozygous genotype calls and dashed lines represent heterozygous genotype calls.

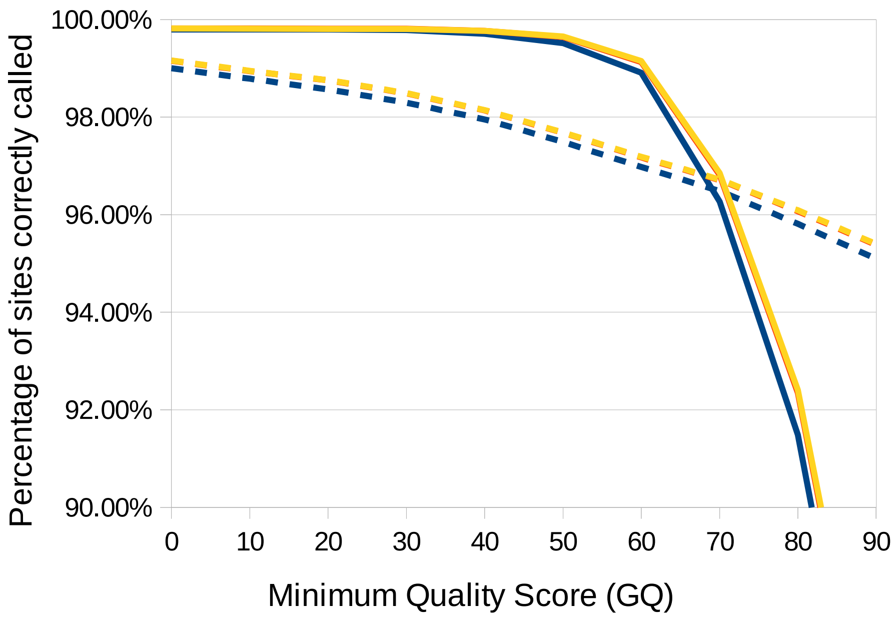

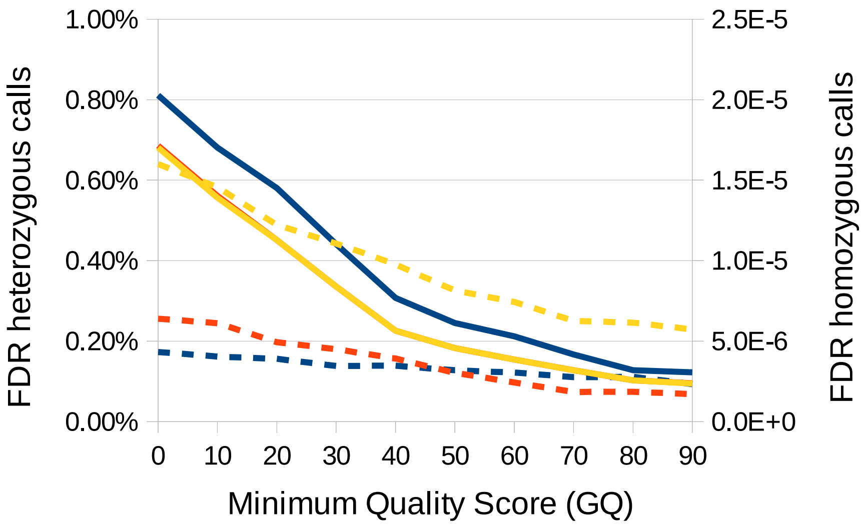

Supplementary Figure 8. ROC curves comparing sensitivity and specificity for (A) heterozygous genotype calls and (B) homozygous genotype calls in small indel genotypes produced by NGSEP (blue), GATK (red), and SAMtools (yellow) using reads aligned with BWA, and NGSEP (green) using reads aligned with Bowtie 2 on the yeast unselected pool

A

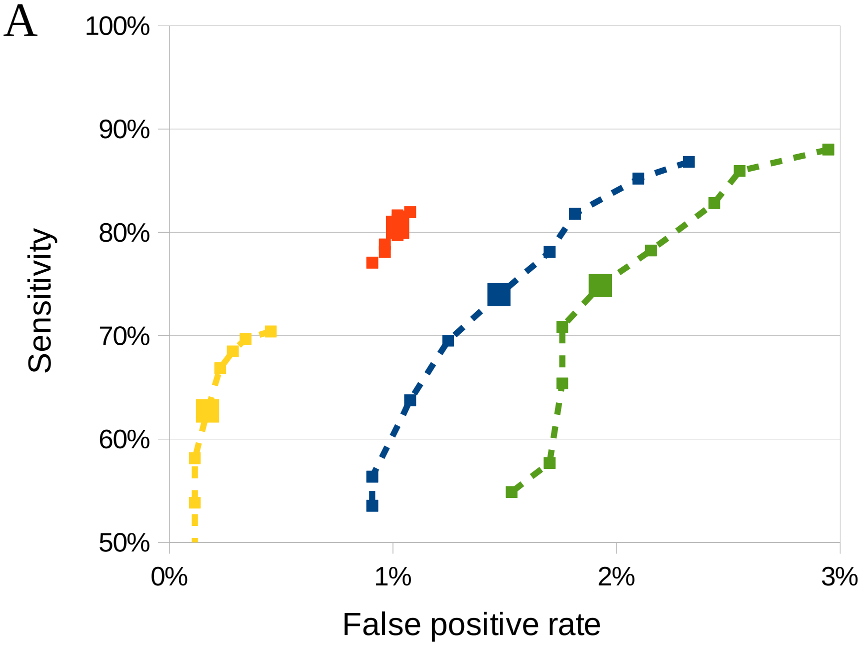

B

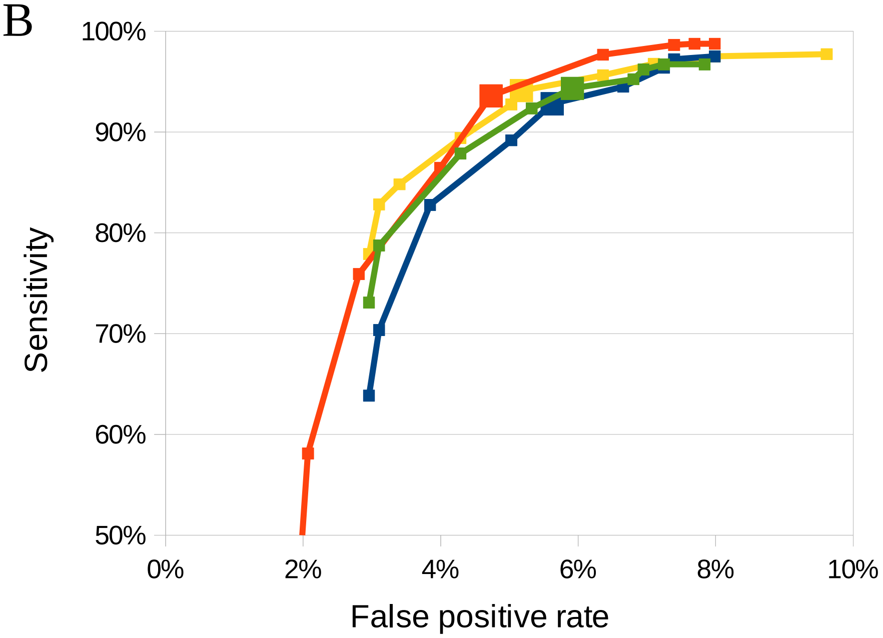

Supplementary figure 9. Differences with the reference genome for the rice sample IR8 as a function of the read position from 5' to 3' (Generated with NGSEP).

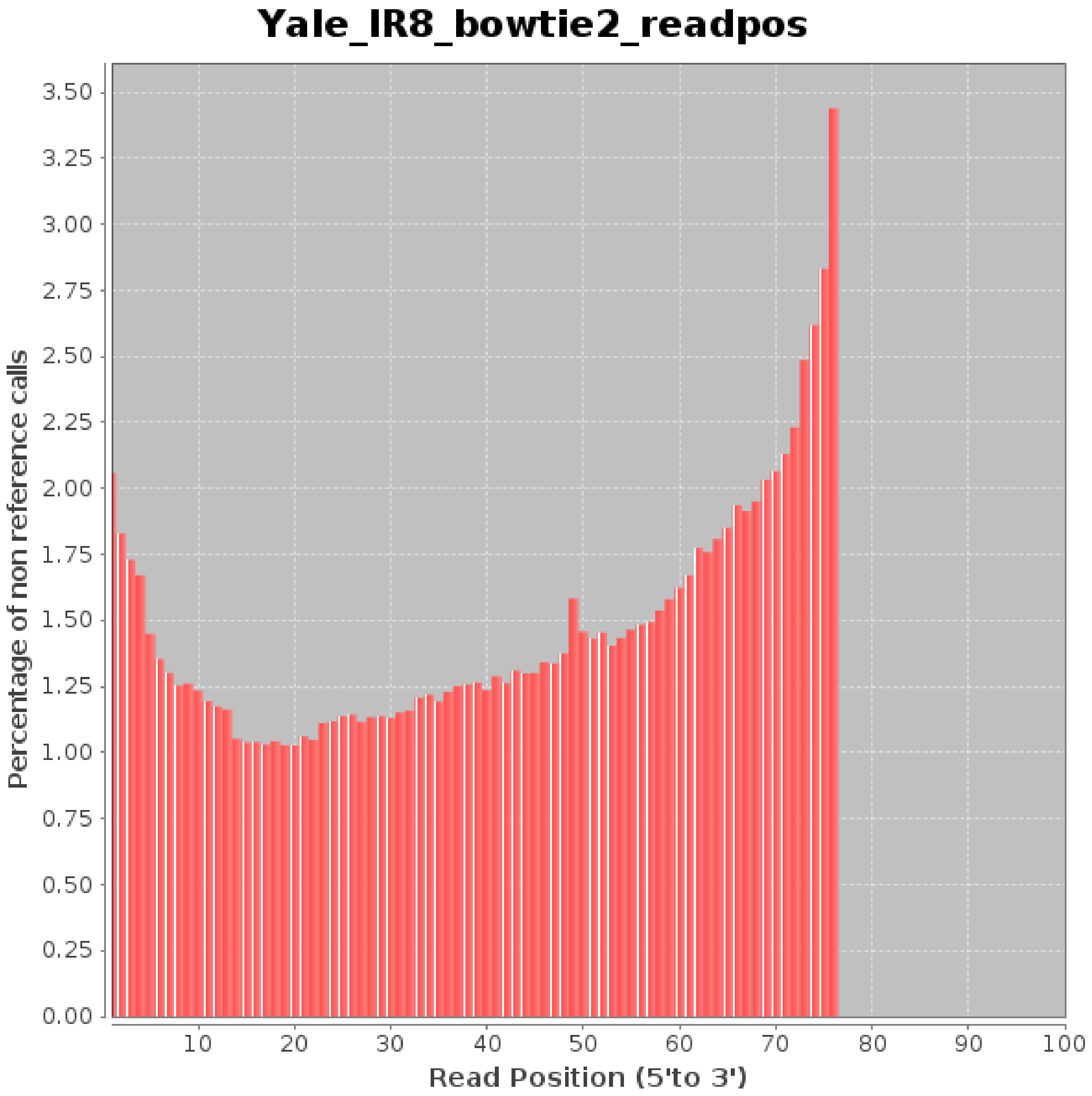

Supplementary figure 10. Percentage of SNVs identified by NGSEP in rice cultivar IR8 that can also be identified by GATK and SAMtools at different minimum quality score thresholds

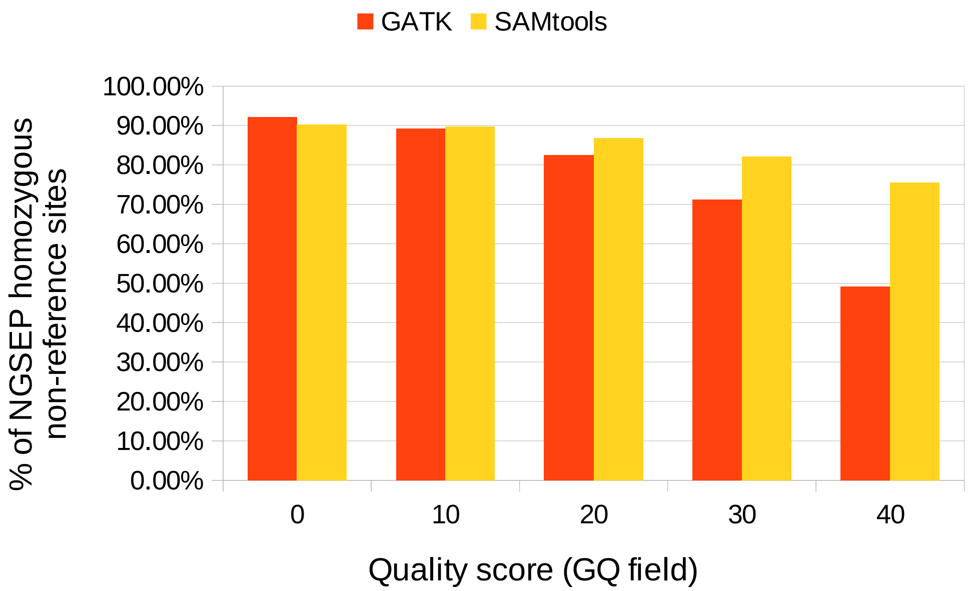

Supplementary figure 11 The defective deletion of the *SD1* gene producing the semi-dwarf phenotype of rice cultivar IR8 could be identified by NGSEP

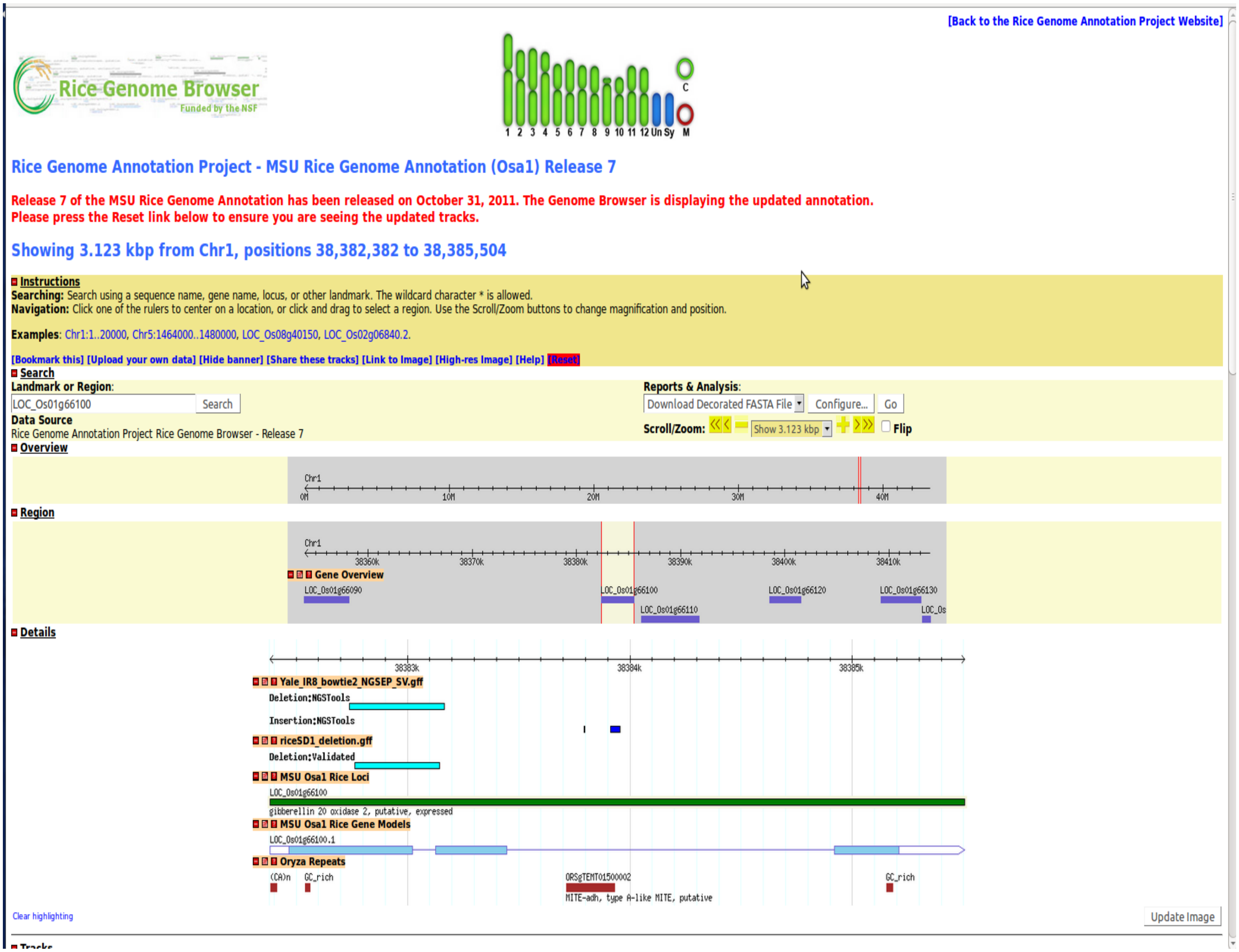

Supplementary script 1. Script used to run the variants detector of NGSEP on the yeast samples. It receives the bam file to analyze, a prefix for the output files and the ploidy of the sample. The last optional parameter was used to assess the behavior of NGSEP under different parameters (Supplementary figures 4 and 5).

```
b=$1;
p=$2;
l=$3;
o=$4;
JAVA="/data/software/jdk1.7.0_40/bin/java -d64 -Xmx4g";
NGSEP="/data/software/NGSEP/NGSToolsApp.jar";
REFERENCE="/data/yeast/reference/sacCer_SGD_refgenome_20110301.fa";
${JAVA} -jar ${NGSEP} FindVariants -noRep -genotypeAll -sampleId ${p} -ploidy ${l} ${o} ${REFERENCE} $
{b} ${p} >& ${p}.log;
awk '{if(substr($0,1,1)=="#" || (length ($4)==1 && length($5)==1)) print $0}' ${p}.vcf > ${p}_snps.vcf;
awk '{if(substr($0,1,1)=="#" || (length ($4)==1 && length($5)>1)) print $0}' ${p}.vcf > $
{p}_triallelic.vcf;
awk '{if(substr($0,1,1)=="#" || length ($4)>1) print $0}' ${p}.vcf > ${p}_indels.vcf;
```

Supplementary script 2. Script used to run the steps of the GATK pipeline on the yeast samples following the best practices described in http://www.broadinstitute.org/gatk/guide/best-practices. The last statement fills the GQ field with the variant quality for calls to the homozygous reference allele for which the GQ field is not provided.

```
p=$1;
REFERENCE=/data/yeast/reference/sacCer_SGD_refgenome_20110301.fa;
KNOWN_SITES=empty.vcf
MARKDUP="/data/software/jdk1.7.0_40/bin/java -Xmx4g -jar /data/software/picard-tools-
1.99/MarkDuplicates.jar"
GATK="/data/software/jdk1.7.0_40/bin/java -Xmx4g -jar /data/software/GenomeAnalysisTK-2.7-2-
g6bda569/GenomeAnalysisTK.jar";
${MARKDUP} AS=true CREATE_INDEX=TRUE I=${p}_sorted.bam o=${p}_sorted_markdup.bam METRICS_FILE=$
{p}_metrics.txt;
${GATK} -T RealignerTargetCreator -I ${p}_sorted_markdup.bam -R ${REFERENCE} -o ${p}_GATK.intervals;
${GATK} -T IndelRealigner -I ${p}_sorted_markdup.bam -R ${REFERENCE} -targetIntervals $
{p}_GATK.intervals -o ${p}_sorted_realigned.bam;
${GATK} -T BaseRecalibrator -I ${p}_sorted_realigned.bam -R ${REFERENCE} -o ${p}_recal_report.grp
-knownSites ${KNOWN_SITES};
${GATK} -T PrintReads -R ${REFERENCE} -I ${p}_sorted_realigned.bam -BQSR ${p}_recal_report.grp -o $
{p}_sorted_recal.bam;
${GATK} -T UnifiedGenotyper -I ${p}_sorted_recal.bam -R ${REFERENCE} -o ${p}_GATK_allsites.vcf
-stand_emit_conf 0.0 -dcov 1000 -glm SNP -out_mode EMIT_ALL_SITES -rf BadCigar;
{GATK} -T UnifiedGenotyper -I ${p}_sorted_recal.bam -R ${REFERENCE} -o ${p}_GATK_Indel.vcf
-stand_emit_conf 0.0 -dcov 1000 -glm INDEL -out_mode EMIT_VARIANTS_ONLY -rf BadCigar;
awk '{if(substr($1,1,3)!="chr" || index($9,"GQ")>0) print $0; else if ($6!=".") printf("%s\t%s\t%s\t
%s\t%s\t%s\t%s\t%s\t%s:GQ\t%s:%d\n", $1,$2,$3,$4,$5,$6,$7,$8,$9,$10,$6)}' ${p}_GATK_allsites.vcf > $
{p}_GATK_allsites_GQall.vcf
```

Supplementary script 3. Script used to genotype the yeast samples using SAMtools following the documentation provided in http://samtools.sourceforge.net/mpileup.shtml. The awk command after the bcftools command fills the GQ field with the variant quality for calls to the homozygous reference allele for which the GQ field is not provided.

```
p=$1;
SAMTOOLSDIR=/data/software/samtools-0.1.19/;
REFERENCE=/data/yeast/reference/sacCer_SGD_refgenome_20110301.fa
touch ${p}_samtools.start;
${SAMTOOLSDIR}/samtools mpileup -uf ${REFERENCE} ${p}_sorted.bam | ${SAMTOOLSDIR}/bcftools/bcftools
view -cg -  | awk '{if($9=="PL")printf("%s\t%s\t.\t%s\t.\t%d\t.\t%s\tGT:GQ\t0/0:%d\n",
$1,$2,$4,$6,$8,$6); else print $0}' > ${p}_samtools.vcf;
grep -v INDEL ${p}_samtools.vcf > ${p}_samtools_allsites.vcf;
awk '{if($3!="." || (substr($8,1,5)=="INDEL" && substr($10,1,3)!="0/0")) print $0}' ${p}_samtools.vcf >
${p}_samtools_indel.vcf;
```

Supplementary script 4. Script used to genotype the yeast samples using VarScan and VarScan2 following the documentation provided in http://varscan.sourceforge.net/germline-calling.html. Awk commands are used to translate to VCF format the output obtained with the perl script “fpfilter.pl”

```
p=$1;
SAMTOOLS=/data/software/samtools-0.1.19/samtools;
JAVA="/data/software/jdk1.7.0_40/bin/java -d64 -Xmx4g";
VARSCANDIR="/data/software/varscan";
REFERENCE="/data/yeast/reference/sacCer_SGD_refgenome_20110301.fa";
touch ${p}_varscan2.start;
for c in I II III IV V VI VII VIII IX X XI XII XIII XIV XV XVI M; do (${VARSCANDIR}/bam-readcount/build/bin/bam-readcount -f ${REFERENCE} ${p}_sorted.bam chr${c}); done > ${p}_varscan2_readcount.txt;
(${SAMTOOLS} mpileup -B -f ${REFERENCE} ${p}_sorted.bam | java -jar ${VARSCANDIR}/VarScan.v2.3.6.jar mpileup2cns --min-coverage 1 --min-reads2 1)  1> ${p}_varscan2.out 2> ${p}_varscan2.log;
perl ${VARSCANDIR}/fpfilter.pl ${p}_varscan2.out ${p}_varscan2_readcount.txt --output-basename ${p}_varscan2_filtered >> ${p}_varscan2.log;
awk -F "\t|:" 'BEGIN{a=log(10)}{if(length($4)==1){g="1/1";if(index("ACGT",$5)<=0)g="0/1";q=-10*log($10)/a;if(q>255)q=255;printf("%s\t%s\t.\t%s\t%s\t%d\tPASS\t.\tGT:GQ:DP\t%s:%d:%d\n", $1,$2,$3,$4,q,g,q,$6)}}' ${p}_varscan2_filtered.pass | cat varscan_head.vcf - > ${p}_varscan2_filtered_snps.vcf
awk -F "\t|:" 'BEGIN{a=log(10)}{if(length($4)>1){g="1/1";if(substr($5,1,1)=="*")g="0/1";q=-10*log($10)/a;if(q>255)q=255;printf("%s\t%s\t.\t%s\t%s\t%d\tPASS\t.\tGT:GQ:DP\t%s:%d:%d\n", $1,$2,$3,$4,q,g,q,$6)}}' ${p}_varscan2_filtered.pass | cat varscan_head.vcf - > ${p}_varscan2_filtered_indels.vcf
touch ${p}_varscan.start;
(${SAMTOOLS} mpileup -B -f ${REFERENCE} ${p}_sorted.bam | java -jar ${VARSCANDIR}/VarScan.v2.3.6.jar mpileup2cns --output-vcf 1 --min-coverage 1 --min-reads2 1 --vcf-sample-list ${p})  1> ${p}_varscan.vcf 2> ${p}_varscan.log;
(${SAMTOOLS} mpileup -B -f ${REFERENCE} ${p}_sorted.bam | java -jar ${VARSCANDIR}/VarScan.v2.3.6.jar mpileup2indel --output-vcf 1 --min-coverage 1 --min-reads2 1 --vcf-sample-list ${p})  1> ${p}_varscan_indels.vcf 2> ${p}_varscan_indels.log;
```

Supplementary script 5. Script used to run the variants detector of NGSEP on the analyzed human samples from the 1000 Genomes Project. It receives the reference genome, the BAM file to analyze, and a prefix for the output files. The last parameter was only used in the high-coverage sample to run NGSEP with the same genome size hard-coded in the available implementation of CNVnator ("-genomeSize 2900000000").

```
r=$1
b=$2;
p=$3;
o=$4;
JAVA="/data/software/jdk1.7.0_40/bin/java -d64 -Xmx8g";
NGSEP="/data/software/NGSEP/NGSToolsApp.jar";
${JAVA} -jar ${NGSEP} FindVariants -noRep -sampleId ${p} ${o} ${r} ${b} ${p} >& ${p}.log;
awk '{if(substr($0,1,1)=="#" || (length ($4)==1 && length($5)==1)) print $0}' ${p}.vcf > ${p}_snps.vcf;
awk '{if(substr($0,1,1)=="#" || (length ($4)==1 && length($5)>1)) print $0}' ${p}.vcf > ${p}_triallelic.vcf;
awk '{if(substr($0,1,1)=="#" || length ($4)>1) print $0}' ${p}.vcf > ${p}_indels.vcf;
```

Supplementary script 6. Script used to run the GATK unified genotyper on the analyzed human samples from the 1000 Genomes Project.

```
b=$1;
p=$2;
REFERENCE=$3;
GATK="/data/software/jdk1.7.0_40/bin/java -Xmx4g -jar /data/software/GenomeAnalysisTK-2.7-2-g6bda569/GenomeAnalysisTK.jar";
${GATK} -T UnifiedGenotyper -I ${b} -R ${REFERENCE} -o ${p}_GATK.vcf -stand_emit_conf 0.0 -dcov 1000;
```

Supplementary script 7. Script used to run SAMtools on the analyzed human samples from the 1000 Genomes Project and on the rice IR8 sample following the documentation provided in http://samtools.sourceforge.net/mpileup.shtml.

```
r=$1;
f=$2;
p=$3;
SAMTOOLSDIR=/data/software/samtools-0.1.19/;
touch ${p}_samtools.start;
${SAMTOOLSDIR}/samtools mpileup -uf ${r} ${f} | ${SAMTOOLSDIR}/bcftools/bcftools view -Nvcg - > $
{p}_samtools.vcf;
grep -v INDEL ${p}_samtools.vcf > ${p}_samtools_snps.vcf;
awk '{if($3!="." || substr($8,1,5)=="INDEL") print $0}' ${p}_samtools.vcf > ${p}_samtools_indel.vcf;
```

Supplementary script 8. Script used to run the variants detector of NGSEP on the rice IR8 sample

```
p=Yale_IR8_bowtie2_NGSEP;
FINDVARIANTS="/data/software/jdk1.7.0_40/bin/java -d64 -Xmx4g -jar /data/software/NGSEP/NGSToolsApp.jar
FindVariants";
${FINDVARIANTS} -h 0.0001 -maxAlnsPerStartPos 2 -minQuality 40 -maxBaseQS 30 -ignore3 4 -sampleId
IR8_NGSEP /data/rice/reference/TIGR7_0/nipponbare_TIGR7.fa Yale_IR8_bowtie2_sorted.bam ${p} >& $
{p}.log;
```

Supplementary script 9. Script used to run the GATK unified genotyper on the rice IR8 sample following the best practices described in http://www.broadinstitute.org/gatk/guide/best-practices.

```
p=$1;
REFERENCE=/data/rice/reference/TIGR7_0/nipponbare_TIGR7.fa;
KNOWN_SITES=/data/bioinformaticsGroup/pipeline/datasets/rice/empty.vcf
MARKDUP="/data/software/jdk1.7.0_40/bin/java -Xmx8g -jar /data/software/picard-tools-
1.99/MarkDuplicates.jar"
GATK="/data/software/jdk1.7.0_40/bin/java -Xmx12g -jar /data/software/GenomeAnalysisTK-2.7-2-
g6bda569/GenomeAnalysisTK.jar";
${MARKDUP} AS=true CREATE_INDEX=TRUE I=${p}_sorted.bam o=${p}_sorted_markdup.bam METRICS_FILE=$
{p}_metrics.txt;
${GATK} -T RealignerTargetCreator -I ${p}_sorted_markdup.bam -R ${REFERENCE} -o ${p}_GATK.intervals;
${GATK} -T IndelRealigner -I ${p}_sorted_markdup.bam -R ${REFERENCE} -targetIntervals $
{p}_GATK.intervals -o ${p}_sorted_realigned.bam;
${GATK} -T BaseRecalibrator -I ${p}_sorted_realigned.bam -R ${REFERENCE} -o ${p}_recal_report.grp
-knownSites ${KNOWN_SITES};
${GATK} -T PrintReads -R ${REFERENCE} -I ${p}_sorted_realigned.bam -BQSR ${p}_recal_report.grp -o $
{p}_sorted_recal.bam;
${GATK} -T UnifiedGenotyper -I ${p}_sorted_recal.bam -R ${REFERENCE} -o ${p}_GATK_snps.vcf
-stand_emit_conf 0.0 -dcov 1000 -hets 0.0001 -glm SNP -out_mode EMIT_VARIANTS_ONLY -rf BadCigar;
${GATK} -T UnifiedGenotyper -I ${p}_sorted_recal.bam -R ${REFERENCE} -o ${p}_GATK_Indel.vcf
-stand_emit_conf 0.0 -dcov 1000 -glm INDEL -out_mode EMIT_VARIANTS_ONLY -rf BadCigar;
```

Supplementary script 10. Java script used to compare VCF files with genotypes for the same sample at different quality scores. To run this script, save it into a file called VariantCallsComparator, compile it and execute it as a java program, including the library NGSToolsApp.jar in the classpath.

```java
import java.io.PrintStream;
import java.util.ArrayList;
import java.util.Arrays;
import java.util.Iterator;
import java.util.List;

import net.sf.ngstools.genome.GenomicRegionComparator;
import net.sf.ngstools.sequences.io.SequenceNameListHandler;
import net.sf.ngstools.variants.CalledGenomicVariant;
import net.sf.ngstools.variants.PhredScoreHelper;
import net.sf.ngstools.variants.io.VCFFileReader;
import net.sf.ngstools.variants.io.VCFRecord;

public class VariantCallsComparator {
	public static void main(String[] args) throws Exception {
		String sequenceNamesFile = args[0];
		String file1 = args[1];
		String file2 = args[2];
		PrintStream out = null;
		int mode = 0;
		double minP = 0;

		SequenceNameListHandler seqHandler = new SequenceNameListHandler();
		GenomicRegionComparator comparator = new
GenomicRegionComparator(seqHandler.loadSequences(sequenceNamesFile));
		VCFFileReader in1 = new VCFFileReader(file1);
		VCFFileReader in2 = new VCFFileReader(file2);
		Iterator<VCFRecord> it1 = in1.iterator();
		Iterator<VCFRecord> it2 = in2.iterator();
		int [][] counts = new int [10][15];
		VCFRecord r1 = it1.next();
		VCFRecord r2 = it2.next();
		while(true) {
			CalledGenomicVariant call1 = r1.getCalls().get(0);
			CalledGenomicVariant call2= r2.getCalls().get(0);
			if(call1.isUndecided() ) {
				if(it1.hasNext()) {
					r1 = it1.next();
					continue;
				}
				else break;
			}
			if(call2.isUndecided() ) {
				if(it2.hasNext()) {
					r2 = it2.next();
					continue;
				}
				else break;
			}
			int cmp = comparator.compare(r1.getVariant(), r2.getVariant());

			int qualI1 = Math.min(9,PhredScoreHelper.calculatePhredScore(1-
call1.getGenotypeProbability())/10);
			int qualI2 = Math.min(9,PhredScoreHelper.calculatePhredScore(1-
call2.getGenotypeProbability())/10);
			int n1 = getGenotypeNumber(call1);
			int n2 = getGenotypeNumber(call2);
			if(cmp<-1) {
				updateCounts(counts,0,qualI1,9+n1);
				if(it1.hasNext()) r1 = it1.next();
				else {
					r1= null;
					break;
				}
			} else if (cmp>1) {
				updateCounts(counts,0,qualI2,12+n2);
```

```
					if(it2.hasNext()) r2 = it2.next();
					else {
						r2 = null;
						break;
					}
				} else {
					int k = 3*n1+n2;
					if(qualI1 <= qualI2) {
						updateCounts(counts,0,qualI1,k);
						updateCounts(counts,qualI1+1,qualI2,12+n2);
					} else {
						updateCounts(counts,0,qualI2,k);
						updateCounts(counts,qualI2+1,qualI1,9+n1);
					}
					boolean done = false;
					if(it1.hasNext()) r1 = it1.next();
					else {
						r1 = null;
						done = true;
					}
					if(it2.hasNext()) r2 = it2.next();
					else {
						r2 = null;
						done = true;
					}
					if(done) break;

				}
			}
			while(r1!=null) {

				CalledGenomicVariant call1 = r1.getCalls().get(0);
				if(!call1.isUndecided()) {
					int qualI1 = Math.min(9,PhredScoreHelper.calculatePhredScore(1-
call1.getGenotypeProbability())/10);
					int n1 = getGenotypeNumber(call1);
					updateCounts(counts,0,qualI1,9+n1);
				}
				if (it1.hasNext()) r1 = it1.next();
				else r1 = null;
			}
			while(r2!=null) {
				CalledGenomicVariant call2 = r2.getCalls().get(0);
				if(!call2.isUndecided()) {
					int qualI2 = Math.min(9,PhredScoreHelper.calculatePhredScore(1-
call2.getGenotypeProbability())/10);
					int n2 = getGenotypeNumber(call2);
					updateCounts(counts,0,qualI2,12+n2);
				}
				if (it2.hasNext()) r2 = it2.next();
				else r2 = null;
			}
			in1.close();
			in2.close();
			printCounts(counts,System.out);

		}

		private static int getGenotypeNumber(CalledGenomicVariant call) {
			if(call.isHeterozygous()) return 1;
			else if (!call.isHomozygousReference()) return 2;
			return 0;
		}

		private static void updateCounts(int[][] counts, int firstRow, int lastRow, int column) {
			for(int i=firstRow;i<=lastRow;i++) {
				counts[i][column]++;
			}
		}
		private static void printCounts(int[][] counts, PrintStream out) {
			for (int i=0;i<counts.length; i++) {
				for(int j=0;j<counts[0].length;j++) {
```

```
                    if(j>0) out.print(" ");
                    out.print(counts[i][j]);
                }
                out.println();
            }
        }
    }
```
